# Targeting PRDX1 impairs acute myeloid leukemic blasts and stem cells by disrupting redox homeostasis

**DOI:** 10.1038/s41419-025-07831-6

**Published:** 2025-08-18

**Authors:** Zhenghao Li, Guangci Liu, Ziren Chen, Keming Li, Zhe Yu, Chao He, Xinyu Ying, Danling Huang, Chengtian Tao, Sajid Khan, Yimeng Wang, Fang-Lin Zhang, Huan Li, Yun Chen, Jingfeng Zhou, Li Yu, Thomas J. Kipps, Yongxian Cheng, Suping Zhang

**Affiliations:** 1 Shenzhen University International Cancer Center, Shenzhen Key Laboratory of Precision Medicine for Hematological Malignancies, Guangdong Key Laboratory for Genome Stability and Human Disease Prevention, Guangdong Provincial Key Laboratory of Chinese Medicine Ingredients and Gut Microbiomics, Marshall Laboratory of Biomedical Engineering, Institute for Inheritance-Based Innovation of Chinese Medicine, Base for International Science and Technology Cooperation: Carson Cancer Stem Cell Vaccines R&D Center, School of Basic Medical Sciences, School of Pharmacy, Shenzhen University Medical School, Shenzhen University, Shenzhen, PR China; 2 Department of Hematology, Shenzhen University General Hospital, Shenzhen, Guangdong PR China; 3 Department of Hematology, Shandong Provincial Third Hospital, Shandong University, Shandong, PR China; 4 Central Laboratory, Jiangsu University Affiliated Fourth Hospital, Zhenjiang, Jiangsu PR China; 5 Department of Clinical Laboratory, Ningbo Medical Centre Lihuili Hospital, Affiliated Hospital of Ningbo University, Ningbo, Zhejiang PR China; 6 School of Chemistry, Chemistry Engineering and Life Sciences, Wuhan University of Technology, Wuhan, Hubei PR China; 7 Department of Immunology, Nanjing Medical University, Nanjing, Jiangsu PR China; 8 Moores Cancer Center, University of California San Diego, La Jolla, CA USA

**Keywords:** Acute myeloid leukaemia, Drug development

## Abstract

Acute myeloid leukemia (AML) is an aggressive hematologic malignancy with a poor prognosis and limited therapeutic options. Leukemic stem cells (LSCs), which drive disease progression and confer resistance to therapy, pose a significant challenge to conventional treatment strategies. In this study, we identified and characterized the inhibitory mechanisms of TH37, a small molecule derived from traditional Chinese medicine, which selectively targets AML blasts and LSCs. Our analyses identified peroxiredoxin 1 (PRDX1), an enzyme that catalyzes the breakdown of hydrogen peroxide (a reactive oxygen species), as the primary molecular target of TH37. We demonstrated that TH37 directly interacts with PRDX1, inhibiting its enzymatic activity and thereby elevating intracellular reactive oxygen species levels in AML cells. PRDX1 was found to be overexpressed in AML, and its expression correlated with poor prognosis and the activation of AML- and cancer-associated pathways. Targeting PRDX1, either through lentiviral short-hairpin RNA-mediated silencing or TH37 treatment, induced apoptosis, reduced colony formation, and impaired the engraftment and growth of AML cells in immunodeficient mouse models. Furthermore, TH37 synergized with conventional chemotherapeutic agent to significantly reduce the viability and colony-forming capacity of AML cells. These findings demonstrate the critical role of PRDX1 in AML pathogenesis and highlight its potential as a key therapeutic target to improve clinical outcomes for AML patients.

## Introduction

Acute myeloid leukemia (AML) is an aggressive malignancy of myeloid cells, characterized by a high relapse rate and often exhibiting intrinsic or acquired drug resistance [1]. Despite recent advances in drug development, AML remains one of the most lethal cancers. This is particularly true for elderly patients (aged >60), whose 5-year overall survival rate can be as low as 10–15%, compared to 20–35% in the general population [2–4]. The standard treatment, known as the 7 + 3 regimen, relies on a combination of anthracycline and cytarabine, and has remained largely unchanged since the 1970s [4].

A major challenge in treating AML involves targeting leukemic stem cells (LSCs), which drive relapse and resistance after chemotherapy or radiation [5–7]. CD34 + CD38− AML cells, the earliest identified LSCs population, demonstrate enhanced colony-formation and disease-initiating activity [8–14]. The frequency of LSCs increases significantly at relapse compared to diagnosis, correlating with a poor prognosis [5]. AML cells often develop multidrug resistance (MDR) through mechanisms such as the overexpression of efflux pumps (e.g., ATP-binding cassette transporters) and *TP53* mutations [15, 16], leading to limited therapeutic options and treatment failure. In addition, conventional chemotherapy drugs not only target leukemic cells but also cause significant toxicity to normal cells, resulting in severe side effects. This restricts a significant proportion of patients from receiving intensive chemotherapy, preventing them from achieving complete remission and increasing the risk of relapse. Therefore, the development of more effective and selective drugs is essential to improve current therapeutic options.

Peroxiredoxin family are ubiquitously expressed and play key roles in regulating reactive oxygen species (ROS) by catalyzing the reduction of hydrogen peroxide (H_2_O_2_) [17]. Peroxiredoxins share a conserved N-terminal cysteine residue (around position 51), which can be oxidized by H_2_O_2_ to form cysteine-sulfenic acid (Cys-SOH) [17]. The role of peroxirodoxin 1 (PRDX1) in cancer remains controversial. In certain cancers, such as breast and lung cancer, PRDX1 has been shown to function as a tumor suppressor by inhibiting c-Myc activity and the PTEN/AKT pathway [17–19]. Whereas other studies demonstrated that PRDX1 promoted tumor progression and metastasis through the activation of c-Jun, AP-1, and epithelial-mesenchymal transition in prostate, pancreatic, and colorectal cancers [19, 20]. While PRDX1 has been reported to suppress FLT3-ITD-induced transformation in AML [21], it also inhibits AML cell differentiation [22]. Thus, the role of PRDX1 in AML has not been fully elucidated.

Malignant cells, including those in AML, typically exhibit elevated basal ROS [23–25]. This is largely attributed to their high proliferative capacity and enhanced cellular respiration, which generate ROS as byproducts, particularly during mitochondrial oxidative phosphorylation(OXPHOS) [26]. ROS, such as H_2_O_2_, also function as signaling molecules that activate cancer-promoting pathways, including Wnt/β-catenin and PI3K/AKT/mTOR [27–29]. Moreover, drug-resistant cancer cells possess higher ROS levels and antioxidant activity than non-resistant cells [30]. However, elevated ROS levels also render cancer cells more vulnerable to ROS-induced apoptosis. To maintain ROS homeostasis, cancer cells often upregulate antioxidant mechanisms [23, 29]. Therefore, triggering ROS production or disrupting antioxidant pathways offers a promising strategy for anticancer therapy [25, 31].

To identify novel therapeutic agents for AML, we screened a small-molecule library derived from traditional Chinese medicine (TCM). TCM is a valuable resource for modern drug discovery, as exemplified by the success of arsenic trioxide (ATO) in treating acute promyelocytic leukemia (APL) [32]. We identified TH37 as a promising candidate exhibiting potent anti-AML activity and low toxicity to normal cells. Mechanistic studies revealed that TH37 acts as a natural inhibitor of PRDX1, whose expression is significantly upregulated in AML and is associated with poor prognosis. Our results demonstrate that PRDX1 is essential for the maintenance of AML cells, both in vitro and in vivo. Furthermore, targeting PRDX1 with lentiviral short-hairpin RNAs (shRNA) or TH37 enhanced the effects of conventional chemotherapeutic drugs, underscoring the potential of TH37 to improve existing treatment strategies for AML.

## Methods

### Cell culture and clinical specimens

The source of each cell line and corresponding culture medium are summarized in Supplementary Table 1. Each cell line identity was verified by short tandem repeat (STR) analysis either prior to distribution or upon receipt. Cells were cultured in DMEM (Hyclone, Logan, UT, USA) or RPMI1640 medium (Gibco, Grand Island, NY, USA) supplemented with 10% fetal bovine serum (FBS) (Hyclone). All cells were maintained at 37 °C in 5% CO_2_ and confirmed as mycoplasma-free through monthly PCR screening.

Bone marrow (BM) or peripheral blood samples were collected from patients with leukemia or healthy donors at Shenzhen University General Hospital who provided written informed consent in compliance with the Declaration of Helsinki and the Institutional Review Board. Mononuclear cells from BM and/or peripheral blood were isolated using Lymphoprep^TM^ (Stemcell Technologies, Vancouver, Canada) density gradient centrifugation.

### Colony formation assay and Drug screening

For colony formation assay of leukemia cell lines, 300–1000 cells per well were suspended in methylcellulose-based growth medium (MethoCult™ H4434, Stemcell Technologies) and plated into 48-well plates. Drug screening was performed at a final drug concentration of 4 μM per well. After 5–7 days of incubation, colonies were counted under an inverted microscope (Nikon, Tokyo, Japan). A >50% reduction in colony formation compared to untreated controls was defined as an effective drug response.

For primary samples, CD34+ cells were enriched from normal or AML samples using EasySep™ Human CD34 Positive Selection Kit II (Stemcell Technologies). Then, 5000–20,000 CD34+ cells were suspended in methylcellulose-based growth medium and plated in 12-well plates. The number of colonies was counted after 7–14 days.

### Extraction and Isolation of TH37

Garcinia resin (600 g Voucher sample: CHYX0629, deposited at the School of Pharmacy, Shenzhen University, PR China) was extracted with 95% ethanol through sonication (3 × 3 L, 1 h each) at room temperature.

The combined extracts were concentrated under reduced pressure to yield a crude residue (390 g). The crude residue was subjected to silica gel column chromatography (200–300 mesh) using a gradient of petroleum ether (PE) and acetone (100:0 to 50:50, v/v), resulting in 10 fractions labeled Fr.A to Fr.J. Fraction Fr.B (2.4 g) was further purified using a Sephadex LH-20 column (MeOH), yielding two subfractions: Fr.B.1 and Fr.B. Fr.B.2 (2.0 g) was separated into seven fractions (Fr.B.2.1 to Fr.B.2.7) using silica gel column chromatography, eluted with PE/ethyl acetate gradient (100:0 to 84:16, v/v). Fr.B.2.5 was fractionated on a Sephadex LH-20 column (MeOH) to produce two subfractions: Fr.B.2.5.1 and Fr.B.2.5.2. Fr.B.2.5.1 (500 mg) was further separated into two portions (Fr.B.2.5.1.1 and Fr.B.2.5.1.2) by preparative HPLC using a gradient of MeOH/H_2_O (80–100%). Fr.B.2.5.1.2 (230.4 mg) was purified by preparative HPLC using a gradient of aqueous methanol (95%) containing 0.05% trifluoroacetic acid, yielding compound TH37 (137.0 mg, *t*_R_ = 14.5 min). The structure of TH37 was elucidated using NMR spectroscopy.

### Western blot analysis

Cells were lysed using RIPA buffer supplemented with a protease inhibitor cocktail (Roche; Cat# 04693132001). Protein concentrations were determined using a BCA assay kit (Thermo Fisher Scientific, Waltham, MA, USA; Cat# 23225). Equal amounts of protein were separated by 10% SDS-PAGE and transferred to PVDF membranes (Millipore, Burlington, MA, USA; Cat# IPVH00010). The membranes were blocked with 5% non-fat milk in TBST (Tris-buffered saline with 0.1% Tween-20) for 1 h at room temperature, then incubated with primary antibodies overnight at 4 °C. Anti-PRDX1 (Thermo Fisher Scientific, 1:1000) and anti-GAPDH (Cell Signaling Technology, 1:2000) were used as primary antibodies, with GAPDH serving as the loading control (Supplementary Table 2). After washing with TBST, the membranes were incubated with HRP-conjugated secondary antibodies (anti-rabbit IgG, Cell Signaling Technology, 1:5000 dilution) for 1 h at room temperature. Protein bands were visualized using an enhanced chemiluminescence detection kit (Thermo Fisher Scientific; Cat# 32106) and imaged with a chemiluminescence detection system.

### Lentivirus infection

For knockdown (KD) of PRDX1, a PLKO.1 plasmid containing shRNA sequences targeting PRDX1 (shPRDX1-1: CAGAUGGUCAGUUUAAAGAUA; shPRDX1-2: CACCTAAGAAACAAGGAGG) or an empty vector (Control) was co-transfected with the packaging plasmids psPAX2 and pMD2.G (Addgene, Cambridge, MA, USA) into 293T cells in a 10-cm dish using polyethylenimine transfection reagent (Polysciences).

Virus-containing media were collected after 48 and 72 h. For infection, the cells were co-cultured in virus-containing medium for 48 h, and 2 µg/ml puromycin was added to select stable PRDX1-KD cells.

For PRDX1 overexpression, CD532A lentivirus vector containing the PRDX1 sequence was used, and the same lentivirus production and infection methods described above were employed.

### In vivo experiments

The IVIS 200 imaging system (Caliper Life Sciences) was used to monitor luciferase-labeled leukemia cell in mice. To create cell line-derived xenograft (CDX) model, 5 × 10^5^ THP-1/luciferase-GFP cells were injected intraperitoneally into 3 to 4 week old NOD-Prkdc^em26Cd52^IL2rg^em26Cd22^/NjuCrl (NCG) mice. The mice were evenly divided into two groups on the following day based on their bioluminescence signals, with one group randomly to be the treatment group. TH37 (2 mg/kg/mouse, diluted in phosphate-buffered saline [PBS] with 3% Tween-80) was administered via the tail vein every 2 days. Bioluminescence signals were monitored every week.

To create the patient-derived xenograft (PDX) model, AML bone marrow mononuclear cells (BMMCs) (5 × 10^6^ cells per mouse) were injected into 1.0 Gy irradiated NCG mice via the tail vein. TH37 was administered using the method described above. AML cell engraftment was monitored according to the percentage of human CD45+ cells in the blood. After sacrifice, BM cells from the femurs were isolated for flow cytometry analysis.

### Flow cytometry analysis

For antibody staining, cells were washed twice with PBS (Hyclone) and resuspended in PBS containing 2% FBS. Antibodies (see Supplementary Table 2) were added at manufacturer-recommended concentrations. After incubation for 30 min at 4 °C, cells were washed twice with PBS. We used 7-aminoactinomycin D (7-AAD, BioLegend, San Diego, CA, USA) and Calcein-Violet (Life Technologies) to stain dead and viable cells, respectively. Fluorescence signals were analyzed using a flow cytometer (Attune™ NxT, Thermo Fisher Scientific). For intracellular staining, cells were fixed with 2% paraformaldehyde and permeabilized using 0.5% Triton X-100. Zombie dye (BioLegend) was used to stain dead cells. A mix of 5% FBS in PBS was used for blocking. Anti-human PRDX1 antibody, conjugated with Alexa Fluor 488 (using the Alexa Fluor 488 conjugation kit, Abcam), was used for staining.

For Annexin-V/7-AAD staining, cells were stained with Annexin-V-FITC (BioLegend) according to the manufacturer’s protocol. After washing with PBS, the cells were incubated in Annexin-V Binding Buffer (BioLegend) with Annexin-V for 15 min. Then 7-AAD was added prior to flow cytometry analysis.

### CCK-8 viability assay

Cells (10,000–15,000) were suspended in 100 µl culture medium per well in a 96-well plate. After 48 h incubation, 10 µl CCK-8 reagent (Beyotime Biotechnology, Shanghai, China) were added to each well and cultured for 1 h. Absorbance was measured at 450 nm using a plate reader, and the IC50 was determined with Graphpad Prism.

### Measuring ROS levels

Cells were rinsed three times with RPMI-1640 medium and then incubated with 5 μM 2’,7’-dichlorodihydrofluorescein diacetate (D6883, Sigma-Aldrich) in RPMI-1640 for 20 min at 37 °C in the dark. After probe loading, cells were treated with 2 μM TH37 for 30–60 min. After treatment, the cells were harvested, and washed three times with ice-cold PBS. Fluorescence intensity was measured using an Attune™ NxT Flow Cytometer (Thermo Fisher Scientific) with excitation/emission wavelengths of 488/530 nm.

### Measuring PRDX1 activity

Recombinant PRDX1 protein purchased from MedChemExpress (NJ, USA) was mixed with hydrogen peroxide substrate with or without TH37. PRDX1 activity was determined by measuring the change in hydrogen peroxide concentration using a hydrogen peroxide assay kit (Elabscience, Wuhan, China) following the manufacturer’s protocol.

### Cellular thermal shift assay

The principle of the cellular thermal shift assay is described in Jafari et al. [33]. PRDX1 protein (0.2 μM) was added into 14 different 200 µL PCR tubes with 2 μM of TH37 or DMSO control. The PCR tubes were heated in a PCR machine at a specified temperature for 3 min, followed by heating at 25 °C for 3 min. The supernatants were mixed with SDS sample buffer and heated at 95 °C for 10 min. Protein samples were then separated by SDS-PAGE and transferred onto a PVDF membrane for immunoblot analysis.

### Enzyme-linked immunosorbent assay

Purified PRDX1 protein in PBS buffer was diluted to a concentration of 1 ng/µL, and 100 µL was used to coat each well of a 96-well ELISA plate stored at 4 °C overnight. The following day, the plate was washed three times with PBST buffer, shaken dry, and blocked with 5% BSA at room temperature for 1 h. Next, 100 µL of TH37-Biotin at various concentrations was added to each well and incubated at room temperature for 1 h, followed by three washes with TBST buffer. Subsequently, 100 µL of peroxidase-conjugated streptavidin (Proteintech, Rosemont, IL, USA) diluted in TBST was added to each well, incubated at room temperature for 1 h, and washed three times with TBST buffer. Finally, 100 µL of tetramethylbenzidine substrate (Sangon Biotech, Shanghai, China) was added and incubated at room temperature in the dark for 20 min, after which 100 µL of 1 M HCl was added to each well for detection.

### Molecular docking analysis

The PRDX1 molecular structure was downloaded from the RCSB Protein Data Bank (https://www.rcsb.org/). Of the eight available structures, 3HY2 was selected for its completeness and highest resolution. The compound’s coordinate set was drawn using ChemDraw 19.0 and converted to PDB format in Chem3D 19.0. AutoDockTools (Version 1.5.6) was used to add hydrogens, assign appropriate atom types, and, if necessary, add charges to both coordinate sets. The possible binding pocket of PRDX1 was predicted using P2Rank. Molecular docking was performed with AutoDock Vina (Version 1.1.2).

### Statistical and clinical database analysis

Sample sizes for in vivo and in vitro experiments were determined based on previously established methods. For experiments involving primary patient samples, the sample size was limited by specimen availability. Patient-derived gene expression and clinical data were obtained from the UCSC Data Hub and cBioPortal. Statistical analyses—including normality tests, determination of statistical differences (e.g., t-test, log-rank test), IC50 determination, and data visualization—were performed using GraphPad Prism (San Diego, CA, USA). The Chou-Talalay combination index was calculated using Compusyn software.

### Single-cell RNA sequencing data analysis

Single-cell RNA sequencing data from GSE120221 (scNBM_1: Bone marrow F, scNBM_2: Bone marrow K) and GSE235063 (scAML_1: PAWHFW, scAML_2: PAWNPG, scAML_3: PAVDYE) were downloaded from the Gene Expression Omnibus (GEO). The data were processed and analyzed using the Seurat R package (Version 5.0). Cell type annotation for each sample was performed using the Azimuth R package (Version 0.5). The datasets were merged, normalized, and scaled prior to performing principal component analysis (PCA). Batch effects were corrected using the Harmony R package (Version 1.21). Finally, PRDX1 expression was visualized on a Uniform Manifold Approximation and Projection plot using the Seurat’s FeaturePlot function.

## Results

### Search for new anti-leukemia drugs in TCM library

**Fig. 1:**
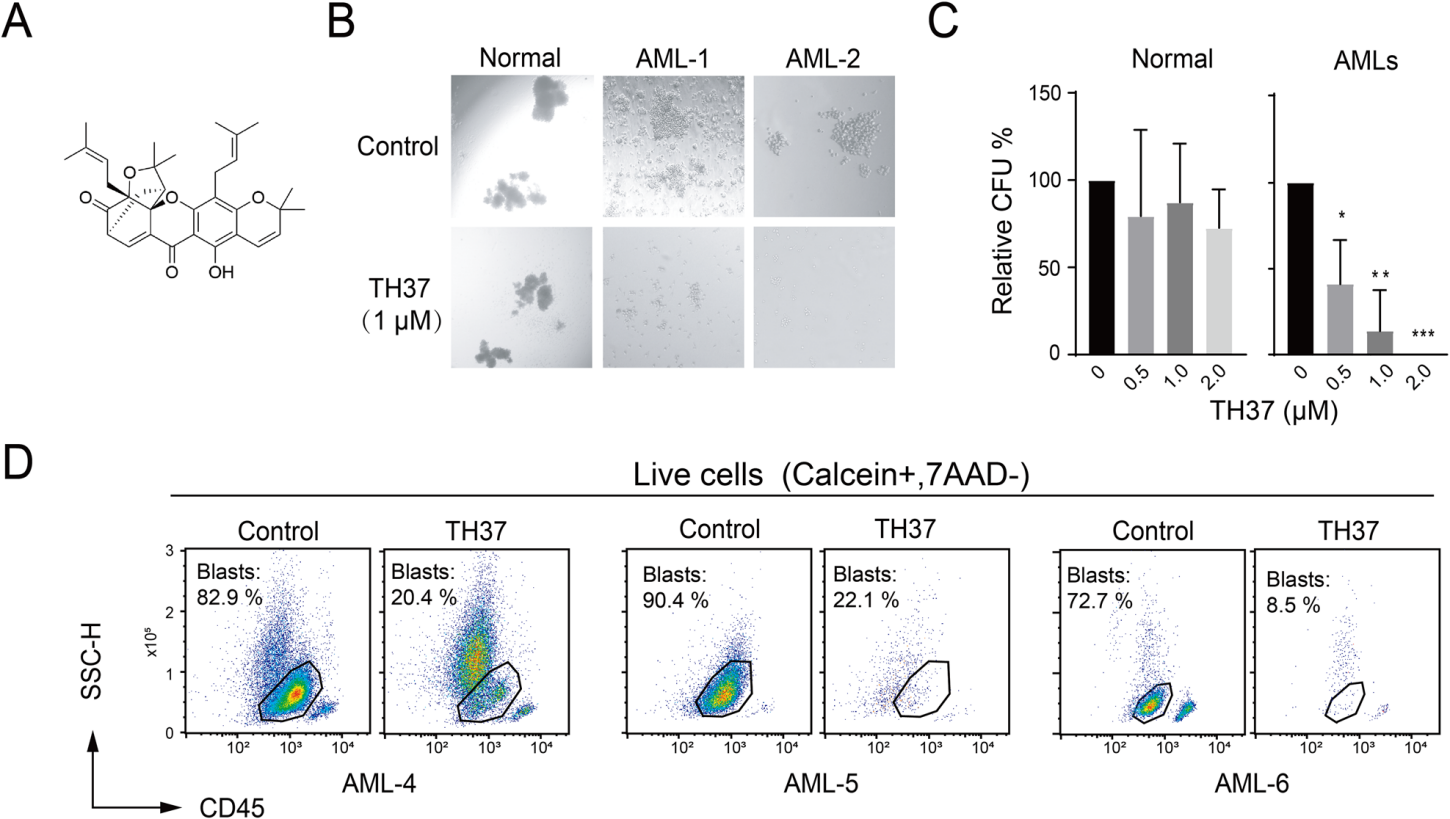
Drug screening and evaluation of TH37 efficacy and safety. **A** Chemical structure of TH37. Results of colony-formation assays using normal CD34+ cells from healthy donors (*n* = 2) and AML patients (*n* = 4) treated with TH37. **B** Representative microscope images. **C** Statistical analysis of the relative colony numbers at different TH37 concentrations. Data are presented as mean ± standard deviation (SD), as assessed by Student’s t-test (*P < 0.05; **P < 0.01; ***P < 0.001 for all figures). **D** Flow cytometry analysis of primary AML treated with TH37 for 18 h demonstrated a significant shift in the blast cell ratio among live cells.

To identify potential new myeloid leukemia inhibitory drugs, we screened a drug library containing 173 compounds derived from TCM, many of which have been traditionally used for cancer treatment. We assessed the impact of these compounds using colony-formation assays of five myeloid leukemia cell lines representing distinct subtypes: THP-1 (AML, MLL-AF9), MV4-11 (AML, FLT3-ITD), HL60 (AML, *TP53*-null), K562 (CML, BCR-ABL1), and KG1a (AML, mutant *TP53*), a LSC-like cell line resistant to conventional chemotherapeutic agents (e.g., doxorubicin and cytarabine) [34]. From the initial screening, we selected the drugs that inhibited colony formation across multiple cell lines in this study (Fig. S1A), and subsequently performed counter-screening using normal CD34+ cells (Fig. S1B). The gamboge-derived compound TH37 (Fig. 1A and Fig. S2) exhibited the lowest toxicity to normal CD34+ cells (Fig. S1B), suggesting favorable selectivity. Furthermore, gamboge has a history of use in TCM for treating tumors. Therefore, we selected TH37 for further investigation.

To further examine the effects of TH37 on primary AML cells, we conducted ex vivo assays using primary cells (clinical information is provided in Table 1). Treatment with TH37 significantly inhibited the colony formation of primary CD34 + AML cells, while showing no significant effects on normal CD34+ hematopoietic stem and progenitor cells (HSPCs) (Fig. 1B, C). Additionally, ex vivo treatment with TH37 markedly reduced the proportion of AML blasts among live cells (Fig. 1D; gating strategy for live cells in Fig. S3).

**Table 1:** Clinical characteristics of patients with AML.

Patient ID	Age;Sex	FAB subtype	Tissue	Status	Genetic abnormality
AML-1	27;F	M4	BM	De novo	FLT3-ITD
AML-2	60;M	M5	BM	De novo	
AML-3	67;M	M2	BM	De novo	
AML-4	73;M	M2	BM	De novo	
AML-5	26;M	M2	BM	De novo	
AML-6	39;M	M4	BM	Relapse	TP53
AML-7	67;M	M2	BM	Relapse	DNMT3A; IDH1; FLT3-ITD
AML-8	45;M	M2	BM	De novo	MLL-AF6; BRAF; KRAS
AML-9	60;F	M5	BM	De novo	FLT3-ITD; NPM1
AML-10	57;F	M5	BM	De novo	+8; DNMT3A; IDH2
AML-11	71;M	M5	Blood	De novo	
AML-12	48;F	M2	Blood	De novo	

*F* female, *M* male, *FAB* French–American–British classification.

### PRDX1 is the primary target of TH37

**Fig. 2:**
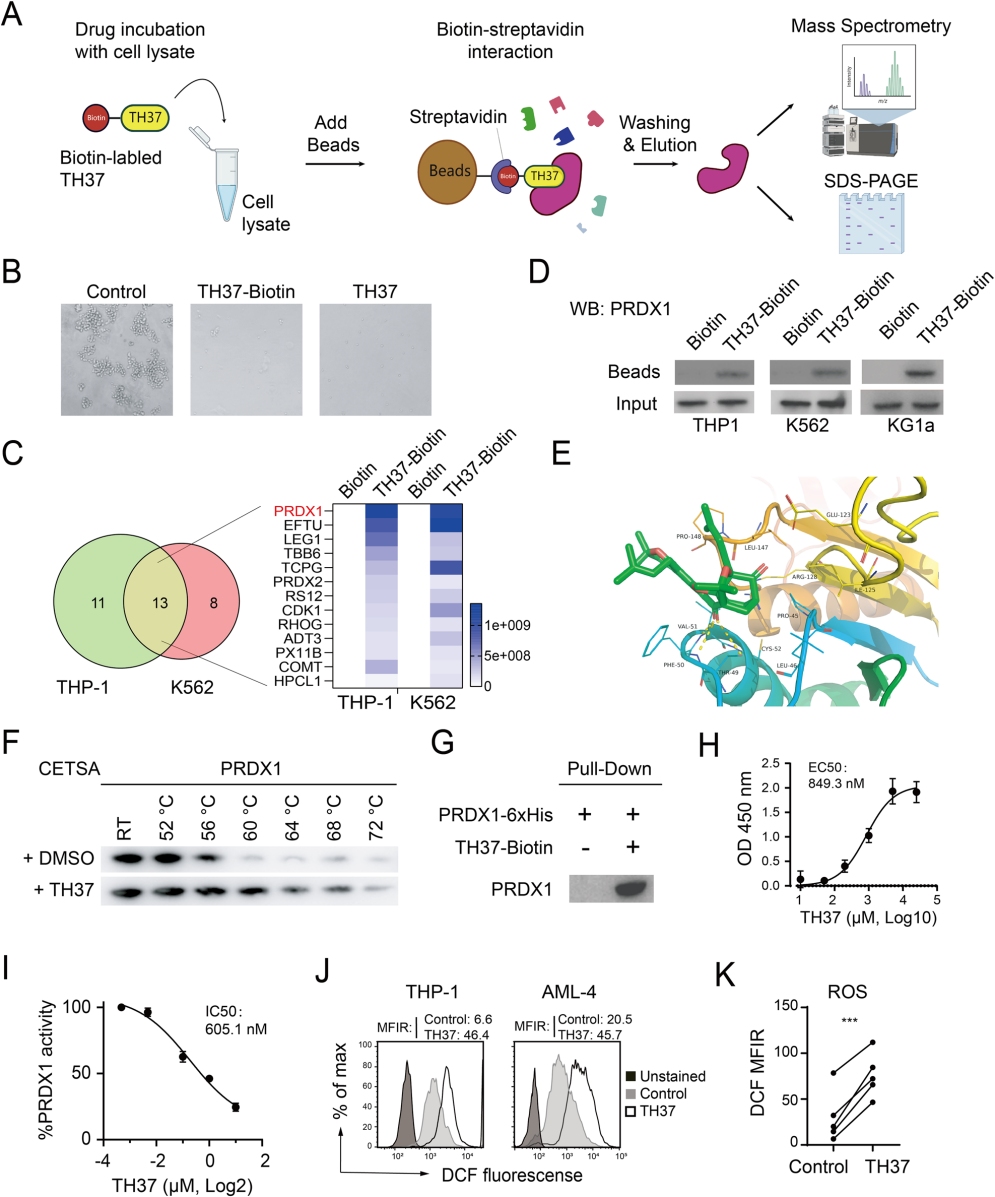
Drug screening and evaluation of TH37 efficacy and safety. **A** Schematic of strategy for identifying potential targets of TH37. **B** Microscope images demonstrating the effect of TH37-biotin on colony formation by K562 cells. **C** MS analysis performed on TH37-biotin co-precipitated proteins from THP-1 and K562 lysates, using N3-biotin as negative control. Gene hits with >50% coverage were selected, and Venn diagrams illustrate the number of hits from the two cell lines, with overlapping genes listed in the right margin. **D** Co-precipitation of PRDX1 and TH37-biotin in various cell lines, as validated by western blotting. **E** Molecular interaction fingerprints of PRDX1 and TH37 (green sticks). The X-ray crystallographic structure of PRDX1 was obtained from the Protein Data Bank (PDB ID: 3HY2). Analysis revealed that TH37 interacts with nine amino acid residues within the binding pocket, forming a hydrogen bond with Thr49 (2.97 Å) and making additional contacts with Phe50 (3.37 Å) and Val51 (3.44 Å). **F** Cellular thermal shift assay results showing the thermal stability of PRDX1 in the presence of TH37. **G** Pull-down assay using purified PRDX1-6xHis and TH37-biotin to validate direct binding. **H** The binding affinity of TH37 for purified PRDX1 protein as measured by ELISA (mean ± SD). **I** Effect of TH37 on the catalytic activity of PRDX1, as assessed with a peroxidase activity assay. **J-K** Cellular ROS levels assessed using the 2,7-dichlorofluorescein (DCF) assay. **J** Cellular ROS levels in representative leukemia cells. Cells were stained with DCF probe and treated with TH37 or DMSO (vehicle control). ROS levels were measured as mean fluorescence intensity ratio (MFIR), defined as the mean fluorescence intensity (MFI) of DCF-stained cells divided by the MFI of unstained controls. **K** Statistical analysis of ROS changes in THP-1, KG1a, K562, HL60 cell lines and primary AML-4 cells treated with TH37 versus DMSO-treated controls was performed using two-tailed paired Student’s *t* test.

To elucidate the molecular mechanism of TH37 activity, we investigated whether TH37 functions through specific protein targets. Thus, we labeled TH37 with biotin (Fig. S4) and used streptavidin beads to capture TH37-associated proteins (Fig. 2A). Biotin labeling was confirmed not to affect the inhibitory activity of TH37 on leukemia cells (Fig. 2B). Through silver staining (Fig. S5) and mass spectrometry (MS) analysis of TH37-biotin co-precipitated proteins, we identified 13 candidate proteins shared by K562 and THP-1 samples (Fig. 2C). Among these, PRDX1 (23 kDa) and EFTU (43 kDa) were significantly more abundant than others. PRDX1, a key regulator of ROS, consistently co-precipitated with TH37 across multiple cell lines (Fig. 2D). In silico docking analysis suggested that TH37 occupied the PRDX1 pocket that contains the active site, Cys52 [17] (Fig. 2E). Moreover, siRNA-mediated knockdown (KD) of PRDX1 significantly impaired cell survival compared to EFTU KD (data not shown), mirroring the effects of TH37. Based on these findings, we focused our subsequent investigations on PRDX1.

To confirm that TH37 directly binds to PRDX1, we performed a cellular thermal shift assay (CETSA) using purified recombinant PRDX1 protein. The results indicated that TH37 significantly stabilized PRDX1 (Fig. 2F), suggesting a direct interaction. This finding was consistent with the pull-down assay results(Fig. 2G). Furthermore, ELISA revealed that TH37 bound to PRDX1 with an EC50 of approximately 800 nM (Fig. 2H). To evaluate the functional impact of TH37 on PRDX1, we assessed the enzymatic activity of PRDX1 using the peroxiredoxin activity assay. As anticipated, TH37 dose-dependently inhibited PRDX1's peroxidase activity (Fig. 2I).

As PRDX1 plays a critical role in the scavenging of H_2_O_2_, a major ROS, we hypothesized that inhibiting PRDX1 with TH37 would lead to the accumulation of cellular ROS, thereby inducing apoptosis. Therefore, we conducted an ROS assay and observed a significant increase in ROS levels in both leukemia cell lines and primary AML cells treated with TH37 (Fig. 2J, K).

### PRDX1 expression is upregulated and associated with poor prognosis in AML

**Fig. 3:**
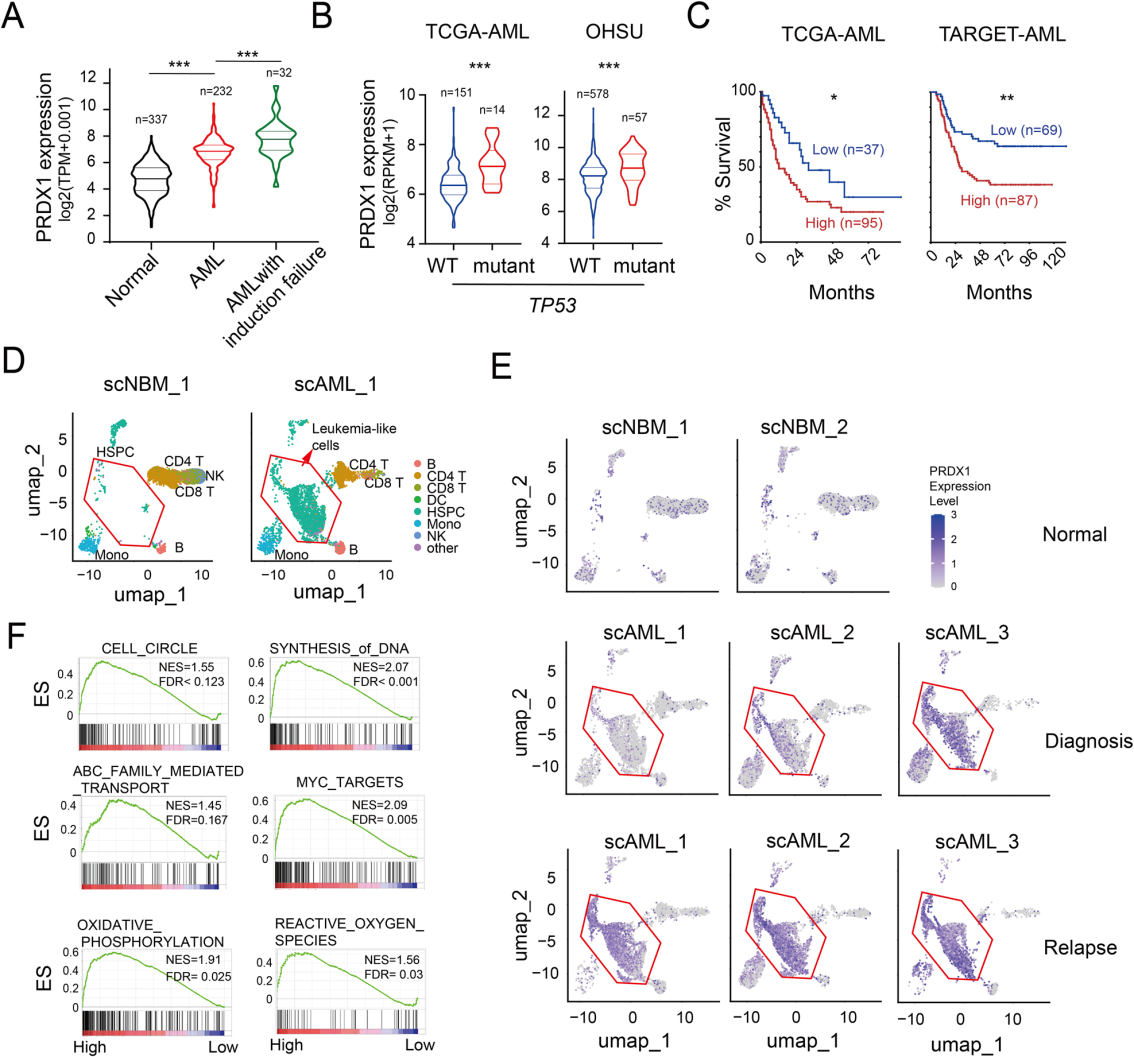
Elevated PRDX1 expression correlates with poor prognosis in AML. **A** The dataset (TCGA-TARGET-Gtex) was downloaded from the UCSC data hub (https://xenabrowser.net/datapages/). A violin plot was generated to visualize PRDX1 mRNA levels in normal white blood cells and AMLs with or without induction therapy failure. Statistical significance was determined using Tukey’s multiple comparisons test. **B** PRDX1 levels in AML samples with wild-type (WT) *TP53* and mutant *TP53*, shown for the TCGA-AML and OHSU datasets. P values were calculated using Mann-Whitney test. **C** Clinical and gene expression data from the TCGA-AML and TARGET-AML datasets were downloaded from UCSC data hub. The association between PRDX1 expression and patient prognosis was assessed using the R2 Kaplan-Meier tool (hgserver1.amc.nl/cgi-bin/r2) with an automatic group cutoff. **D-E** PRDX1 expression levels assessed at the single-cell level using scRNA-seq data from normal BM (scNBM_1 and scNBM_2) and AML BM (scAML_1, scAML_2 and scAML_3). **D** Strategy to identify leukemia-like populations. Leukemia-like cells, overrepresented in AML samples, were classified as HSPCs using Azimuth cell type annotation. **E** PRDX1 expression levels in normal and AML BM samples at diagnosis and relapse. **F** TARGET-AML expression matrix was divided into PRDX1-high and PRDX1-low expression groups based on the median expression level of PRDX1. Gene set enrichment analysis plots were generated to analyze pathways associated with PRDX1 expression.

To investigate the role of PRDX1 in myeloid leukemia, we analyzed publicly available datasets. PRDX1 expression was significantly elevated in myeloid leukemia cells compared to normal white blood cells, with even higher levels observed in samples from patients who failed to respond to induction therapy (Fig. 3A). Additionally, increased PRDX1 expression was correlated with *TP53* mutations and was associated with worse clinical outcomes in AML patients (Fig. 3B, C), suggesting a potential role for PRDX1 in disease progression and treatment resistance.

To further characterize PRDX1 expression in primary AML cells, we analyzed scRNA-seq data from publicly available datasets. The integration of AML and normal BM samples revealed distinct leukemia-like cell populations that were significantly more abundant in AML BM samples than normal control samples. These populations were classified as HSPCs using the Azimuth cell type annotation tool (Fig. 3D). PRDX1 expression was markedly higher in these leukemia-like cells at diagnosis or relapse than in normal BM cells (Fig. 3E).

Gene set enrichment analysis (GSEA) demonstrated a positive correlation between high PRDX1 expression and pathways associated with the cell cycle, cancer, and drug resistance, including DNA synthesis, MYC targets, and ATP-binding cassette(ABC) transporters (Fig. 3F). Additionally, OXPHOS, an ATP-producing metabolic process that is highly utilized by AML CD34 + CD38− LSCs [35], was significantly more active in samples with high PRDX1 expression, highlighting PRDX1's potential role in AML pathogenesis. Furthermore, high PRDX1 expression was associated with the increased expression of ROS-related genes, aligning with the observation that cancer cells typically exhibit elevated ROS levels accompanied by enhanced antioxidant activity. Consistent with this, AML cells demonstrated significantly increased ROS levels (Fig. S6) and elevated PRDX1 expression (Fig. 3A).

### Targeting PRDX1 induced apoptosis in AML cells

**Fig. 4:**
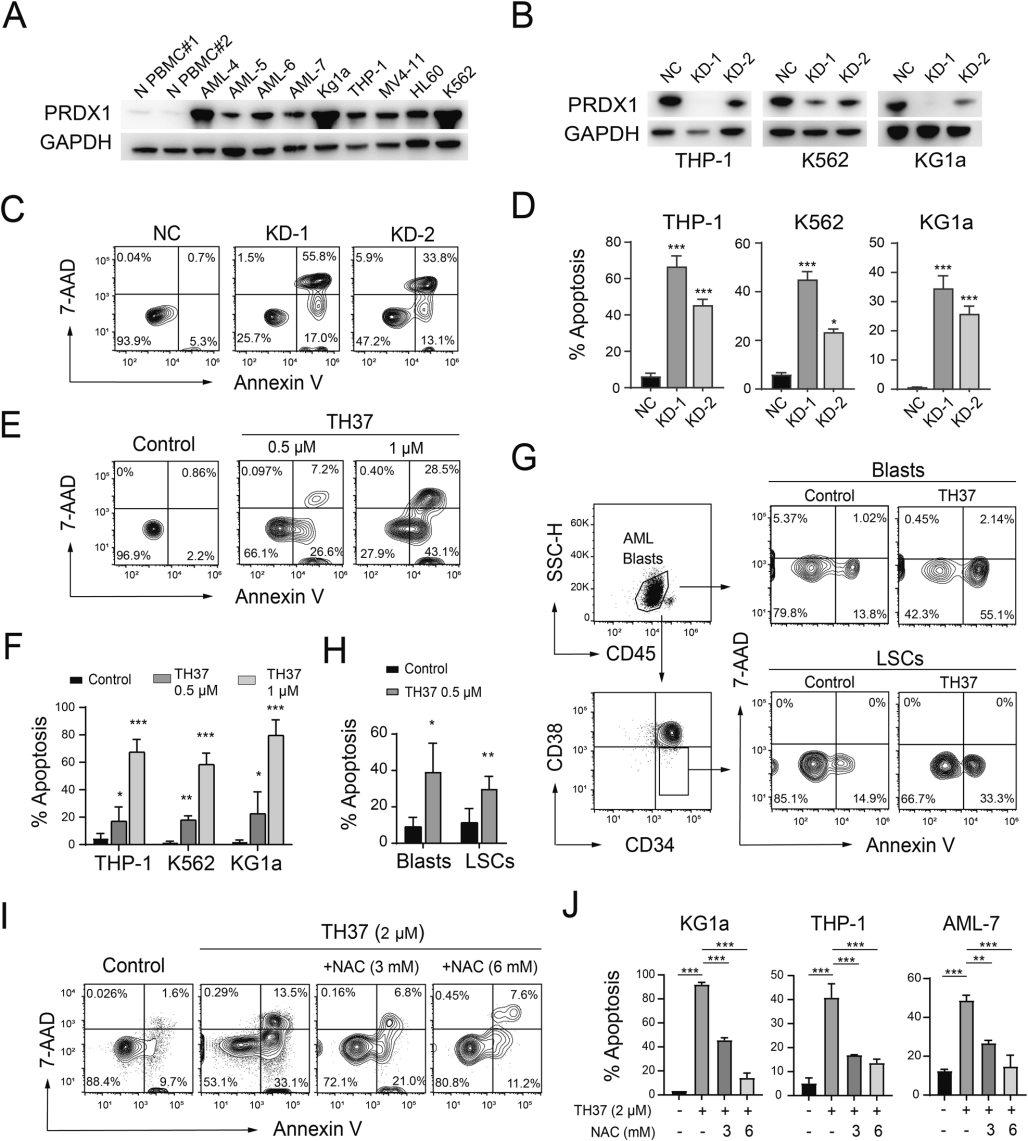
Targeting PRDX1 promoted apoptosis in AML cells. **A** Immunoblot analysis of PRDX1 protein levels in AML cells compared to PBMC lysates from healthy donors. **B-D** Effects of PRDX1 KD in leukemia cells. **B** Immunoblot analysis of PRDX1 levels in leukemia cells infected with lentivirus for delivering PRDX1 shRNAs (KD-1 or KD-2) or negative control shRNA (NC). **C** Representative contour plots from flow cytometry analysis. **D** Flow cytometry analysis of apoptosis by Annexin-V/7-AAD staining. **E-F** Leukemia cell lines were treated with TH37 or DMSO control for 12 h, followed by Annexin-V/7-AAD staining. **E** Representative contour plots from flow cytometry analysis. **F** Statistical analysis of apoptosis in each cell line. **G-H** Flow cytometry analysis of apoptosis in TH37-treated primary AML BMMCs, focusing on blasts (CD45 dim, SSC-low) and LSC-enriched cells (CD45 dim, SSC-low, CD34+, CD38−). **G** Representative Flow cytometry analysis results. **H** Statistical analysis showing the mean ± SD of apoptotic cell percentages in primary AML samples (AML-4, AML-5, and AML-8), as assessed via paired Student’s *t* test. NAC (Selleck Chemicals, Houston, TX, USA) was added to TH37-treated AML cells. **I-J** Apoptosis was assessed using Annexin-V/7-AAD staining in the presence or absence of NAC. **I** Representative flow cytometry plots. **J** Statistical analysis confirmed that NAC significantly attenuated TH37-induced apoptosis across each cell type (mean ± SD; *n* = 3). Significance was assessed using a two-tailed Student’s *t* test.

Western blot analysis confirmed that PRDX1 protein levels were elevated in AML cells compared to normal PBMCs (Fig. 4A). To further investigate PRDX1 expression in LSCs, we performed intracellular staining on primary samples. Consistent with the western blot data, PBMCs exhibited significantly lower PRDX1 levels than LSCs and non-LSC leukemic cells (Fig. S7A, B). LSCs exhibited higher PRDX1 expression levels (median MFIR = 31.5) than non-LSCs (median MFIR = 30.9) in patients with AML (*n* = 6), although the difference was not statistically significant (Fig. S7C).

To investigate the functional role of PRDX1, we employed shRNAs to knock down PRDX1 expression in various cell lines (Fig. 4B). Using AnnexinV and 7-AAD staining to detect early and late apoptosis, respectively, we found that PRDX1 depletion significantly increased apoptosis in AML cells (Fig. 4C, D). Notably, PRDX1 KD also led to increased ROS levels (Fig. S8), consistent with the effects observed upon TH37 treatment, supporting the role of PRDX1 in maintaining redox balance and cell survival.

Similarly, a 500-1000 nM dose of TH37 induced a strong increase in apoptosis in leukemia cell lines (Fig. 4E, F). Consistent with this, CCK-8 assays revealed that the IC50 of TH37 for most myeloid leukemia cell lines was below 1 µM (Table 2). In primary AML samples, TH37 effectively induced apoptosis not only in bulk/blast cells (SSC-low, CD45-dim) but also LSC-enriched populations (SSC-low, CD45-dim, CD34+, CD38−) (Fig. 4G, H).

**Table 2:** IC50 values of TH37 in leukemia cell lines.

Cells	IC50 (μM)
THP-1	0.58
KG1a	0.28
HL60	0.48
MV4-11	0.34
K562	1.02
Kasumi-1	0.62
SKNO-1	0.30

To confirm that TH37 induces apoptosis via ROS generation, we performed a rescue assay using the antioxidant N-acetylcysteine (NAC). Treatment with NAC significantly reduced TH37-induced apoptosis in both AML cell lines and primary cells (Fig. 4I, J), indicating that ROS mediate TH37-triggered apoptosis.

We hypothesized that elevating PRDX1 levels would mitigate ROS accumulation, thereby reducing the effects of TH37, while decreasing PRDX1 expression would enhance its efficacy. Consistent with this hypothesis, PRDX1 overexpression decreased AML cell sensitivity to TH37, while KD of PRDX1 increased it (Fig. S9).

These findings support the notion that PRDX1 plays a critical role in modulating ROS levels and cellular responses to TH37, further highlighting its potential as a therapeutic target in AML.

### Targeting PRDX1 suppressed leukemia engraftment/cell growth in vivo and prolonged mouse survival

**Fig. 5:**
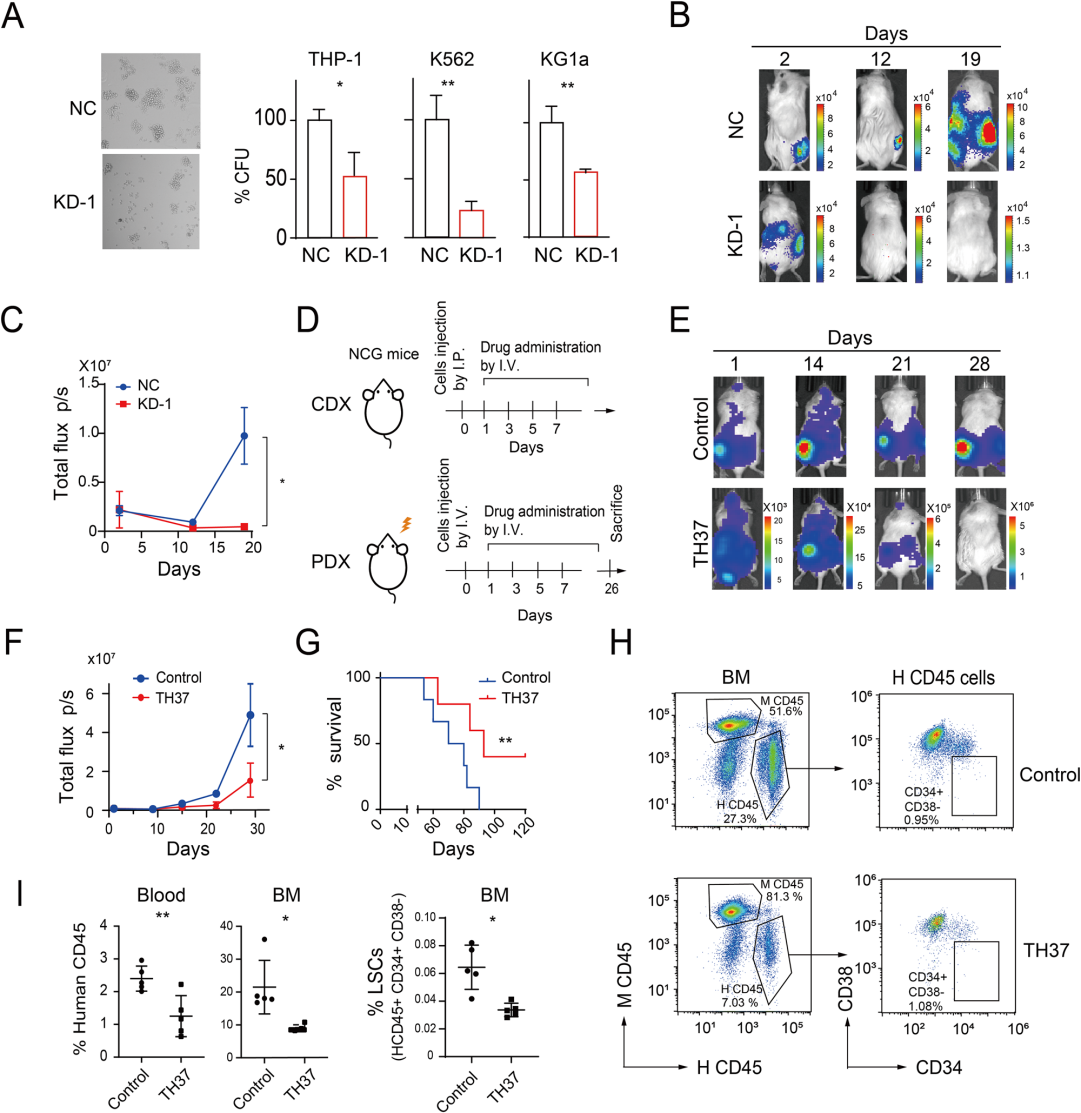
Targeting PRDX1 suppressed leukemia engraftment and growth in mice. **A** Effects of PRDX1 KD on colony formation activity. Left panel: Representative microscope images of colony formation in THP-1 cells. Right panel: Statistical analysis showing the mean ± SD of relative percentage of CFUs for each cell line, as assessed via Student’s *t* test. **B-C** NGC mice were intravenously injected with PRDX1-KD (*n* = 5) or control (*n* = 5) luciferase-labeled THP-1 cells. **B** Representative bioluminescence images of mice at the indicated time points. Photon flux (photons per second, p/s) is indicated by color. **C** Line graph depicting the mean ± standard error of the mean (SEM) of bioluminescence signal intensity for each group of mice. **D** Drug treatment scheme for TH37 in CDX and PDX models. **E-G** Result of CDX model. **E** Representative bioluminescent images of mice at the indicated time points following THP-1 cell injection. **F** Mean ± SEM of bioluminescent signal in Control (*n* = 6) and TH37-treated (*n* = 5) groups at various time points. **G** Kaplan–Meier analysis of survival in each group of mice. Log-rank test was used for statistical analysis. **H-I** Efficacy of TH37 in AML PDX model. **H** Gating strategy to assess the ratio of human CD45+ (H CD45) or CD34 + CD38− cells in mouse BM or blood. **I** Comparison of disease burden between the two groups, as measured by the frequency of AML cells in blood or BM, shown as the percentage of H CD45+ or human CD45 + CD34 + CD38− cells among total human and mouse CD45+ (M CD45) cells.

To further evaluate the role of PRDX1 in LSCs, we examined the impact of PRDX1 depletion on colony-forming activity. Stable PRDX1-KD cells showed a significant reduction in colony-forming ability (Fig. 5A). Consistent with these findings, in vivo experiments demonstrated that PRDX1 KD in luciferase/THP-1 cells significantly reduced engraftment and delayed disease progression in mice (Fig. 5B, C), further supporting the importance of PRDX1 in LSC maintenance and function.

To determine whether TH37 treatment can recapitulate these effects, we utilized both a CDX model and a PDX model (Fig. 5D). In the CDX model, THP-1/GFP-luciferase cells were injected intraperitoneally into NCG mice. TH37 treatment significantly suppressed the growth of leukemia cells (Fig. 5E, F) and improved survival in the treated group (Fig. 5G). We did not observe significant changes in body weight (Fig. S10A) or observable adverse effects in TH37-treated mice, suggesting the treament had a favorable safety profile. In the PDX model, primary AML BMMCs were injected intravenously into irradiated mice. Engraftment and disease burden were assessed by measuring the proportion of human CD45+ cells in the blood and BM. TH37 treatment significantly reduced the ratio of AML cells in both the BM and blood of mice, as well as the proportion of CD34 + CD38− LSC-enriched cells in the BM (Fig. 5H, I).

To further assess the potential toxicity of TH37, we performed 28-day toxicity studies in wild-type (C57BL/6) mice, including a high-dose group (twice the dose given in earlier experments). Histopathological examination (H&E staining) of the heart, lungs, liver, and kidneys revealed no significant abnormalities (Fig. S10B). Consistent with this, serum biochemical analysis showed no significant changes in most parameters, except for a reduction in creatinine (CRE) levels in the high-dose group (Fig. S10C). However, a slight increase was observed in the alanine aminotransferase/aspartate aminotransferase ratio, suggesting the potential for hepatotoxicity with prolonged treatment or higher doses of TH37 (Fig. S10C).

### PRDX1 targeting treatments had synergistic effects with conventional chemotherapy drug

**Fig. 6:**
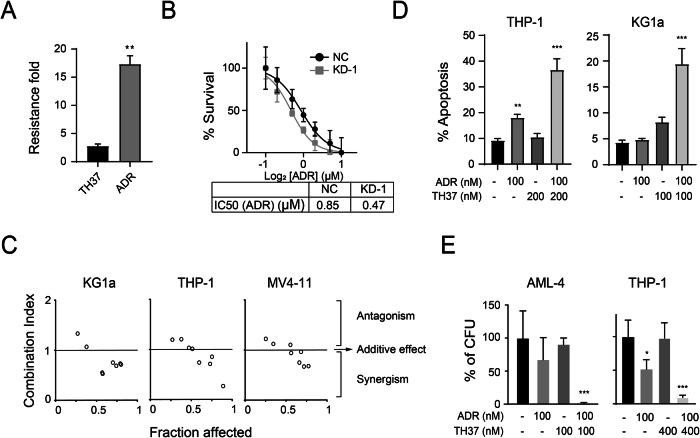
Targeting PRDX1 in AML cells enhanced sensitivity to chemotherapy drug. **A** Comparison of the fold-change in resistance of K562-ADR cells to ADR and TH37, defined as the ratio of IC50(K562-ADR) to IC50(K562) for each drug. **B** Measurement of IC50 for ADR in stable PRDX1-KD or NC KG1a cells using the CCK-8 assay. **C** Analysis of combination effects of TH37 and ADR on leukemia cell lines in the CCK-8 assay. The Chou–Talalay combination index (CI) was calculated. **D** Effect of the TH37 and ADR combination on apoptosis. **E** Effect of the TH37 and ADR combination on colony-formation of THP-1 cell line or primary CD34 + AML cells. Statistical significance was analyzed using one-way ANOVA for multiple comparisons. Data are presented as mean ± SD.

Cross-resistance or MDR is a major cause of drug treatment failure. To examine whether TH37 would remain effective, we assessed its effect on the multidrug-resistant cell line K562-ADR, a K562 variant that overexpresses the P-glycoprotein (MDR1) efflux transporter, which confers MDR [36]. We compared the fold-change in resistance of K562-ADR cells to TH37 and Adriamycin (ADR) relative to K562 cells, and found that, while resistance to ADR was in excess of 17-fold greater, resistance to TH37 was around 2.5 folds higher (Fig. 6A), indicating a low degree of cross-resistance between TH37 and the conventional chemical drug.

Given that elevated ROS levels have been reported to impair drug resistance mechanisms [37], we investigated whether targeting PRDX1 would enhance drug sensitivity. PRDX1-KD in AML cells significantly reduced their IC50 for ADR, indicating that PRDX1 depletion sensitized the AML cells to the conventional chemotherapeutic drug (Fig. 6B).

To examine whether TH37 treatment elicits similar effects, we evaluated the combined effects of TH37 and ADR in CCK-8 viability assays. The Chou-Talalay combination index (CI) [38] for TH37 and ADR were found to be less than 1, indicating a synergistic interaction occurred between the two drugs (Fig. 6C). To validate these findings, we investigated the combined effects of the drugs on apoptosis and colony-formation. While low-dose treatments of TH37 or ADR alone had limited effects on apoptosis and colony formation, in combination, they significantly enhanced apoptosis and suppressed colony formation (Fig. 6D, E). These results highlight the potential of combining TH37 with conventional chemotherapy agents to overcome drug resistance and enhance anti-leukemic efficacy.

## Discussion

The development of highly effective and selective drugs to target LSCs is crucial for minimizing relapse and improving the long-term survival of patients with AML. Several TCM agents have demonstrated success in modern clinical applications. For instance, ATO, a TCM-derived compound used in the treatment of APL/M3-AML, has dramatically increased survival rates from below 30% to over 90% [39, 40]. However, the majority of TCM compounds remain underexplored. Inspired by these facts, we conducted drug screenings using a TCM-derived small-molecule library and identified TH37 as a promising therapeutic candidate for AML treatment. TH37 was highly efficient in eliminating both blast cells and LSCs while exhibiting low toxicity to normal HSPCs. TH37’s efficacy was validated in AML mouse models, including PDX, without significant adverse effects. Moreover, the administration of TH37 at higher doses did not induce significant histopathological abnormalities in the major organs of mice. However, our findings suggest that prolonged treatment or further dose escalation may lead to hepatotoxicity. Additionally, decreased CRE levels were observed in the high-dose group, the underlying mechanism of which remains unclear and requires further investigation.

Our study identified PRDX1 as the primary target of TH37. PRDX1 plays distinct roles in tumorigenesis, acting as both a promoter and a suppressor depending on the cancer type [19, 20]. In this study, we found that elevated PRDX1 expression was associated with poor prognosis and malignant characteristics, such as *TP53* mutations, which are linked to poor responses to chemotherapy [41]. Further experimental results demonstrated that PRDX1 is required for maintaining colony-formation activity, a feature recently identified as a strong indicator of poor prognosis [42]. In consistent findings, the KD of PRDX1 in AML cells impaired their engraftment and growth in mice, underscoring the critical role of this enzyme in AML maintenance.

Highly proliferative cancer cells undergo metabolic reprogramming to meet their large energy demands. Glycolysis and OXPHOS are two major sources of ATP. LSCs predominantly rely on OXPHOS, whereas normal hematopoietic stem cells rely on glycolysis and thus exhibit low ROS levels [35, 43]. ROS are both metabolic byproducts and active effectors that promote cellular proliferation [24]. AML driver mutations, including RAS and FLT3-ITD, are associated with high levels of ROS production [44]. Because excessive ROS trigger apoptosis, cancer cells often exhibit elevated antioxidant enzyme activity to limit ROS [23]. Consistent with these findings, we found that PRDX1 is closely linked to cell cycle, OXPHOS, and ROS pathways in AML, and targeting PRDX1 promoted ROS levels and induced apoptosis. The selectivity of TH37 may be attributed to the high ROS levels in cancer cells, which make them more prone to reaching cytotoxic thresholds [31, 45]. Moreover, our findings align with the observation that promoting ROS can impair MDR, potentially by oxidizing NADH to NAD+ and thereby reducing ATP availability for efflux pump activity [46].

In a notable colon cancer study, PRDX1 was identified as a target of Celastrol, a promising natural product with antitumor properties. Similarly to TH37, celastrol can promote the production of ROS in cancer cells [47]. Another study revealed that H7, a different PRDX1 inhibitor, can promote differentiation in AML cells [22]. The differential effects of TH37 and H7 may result from their distinct IC50 values for inhibiting PRDX1 activity: H7 has an IC50 of 7.8 µM, more than 10 times higher than that of TH37 (0.6 µM). This difference in potency likely results in varying levels of ROS elevation and subsequent cell fate differences, as moderate increases in ROS production can promote differentiation signaling, whereas highly excessive ROS can trigger apoptosis [25].

Drug combination strategies are commonly used to enhance the efficacy of therapeutics. In our study, we observed that TH37 enhanced the effects of the chemotherapy drug ADR on AML cells, which we attributed to multiple mechanisms. First, targeting PRDX1 enhanced the sensitivity of AML cells to chemotherapy drug, and similar findings have been observed in breast cancer [48]. Second, TH37 exhibited low cross-resistance to conventional chemotherapy drug, suggesting it may help overcome tumor heterogeneity by effectively targeting drug-resistant subpopulations within tumors. However, the effects of combining TH37 with other antioxidant agents or targeted drugs, as well as the safety and efficacy of these combinations in in vivo models, requires further investigation.

Taken together, our findings underscore the critical role of PRDX1 in AML blast and LSC maintenance. Targeting PRDX1 has the potential to be an effective therapeutic strategy to improve the treatment of de novo and relapsed/refractory AML.

## Supplementary information

Supplementary Data

Original WB data

## Data Availability

The data that support the findings of this study are available from the corresponding author upon reasonable request.

## References

[1] de Lima M, Roboz GJ, Platzbecker U, Craddock C, Ossenkoppele G. AML and the art of remission maintenance. Blood Rev. 2021;49:100829. 33832807 10.1016/j.blre.2021.100829

[2] Kantarjian H, Kadia T, DiNardo C, Daver N, Borthakur G, Jabbour E, et al. Acute myeloid leukemia: current progress and future directions. Blood Cancer J. 2021;11:41. 33619261 10.1038/s41408-021-00425-3 PMC7900255

[3] Rowe JM. Perspectives on current survival and new developments in AML. Best Pr Res Clin Haematol. 2021;34:101248. 10.1016/j.beha.2021.101248 33762103

[4] Stanchina M, Soong D, Zheng-Lin B, Watts JM, Taylor J. Advances in acute myeloid leukemia: recently approved therapies and drugs in development. Cancers. 2020;12. 10.3390/cancers12113225 PMC7692236 33139625

[5] Lane SW, Gilliland DG. Leukemia stem cells. Semin Cancer Biol. 2010;20:71–6. 20026405 10.1016/j.semcancer.2009.12.001

[6] Pollyea DA, Gutman JA, Gore L, Smith CA, Jordan CT. Targeting acute myeloid leukemia stem cells: a review and principles for the development of clinical trials. Haematologica. 2014;99:1277–84. 25082785 10.3324/haematol.2013.085209 PMC4116825

[7] Pollyea DA, Jordan CT. Therapeutic targeting of acute myeloid leukemia stem cells. Blood. 2017;129:1627–35. 28159738 10.1182/blood-2016-10-696039

[8] Arnone M, Konantz M, Hanns P, Paczulla Stanger AM, Bertels S, Godavarthy PS, et al. Acute myeloid leukemia stem cells: the challenges of phenotypic heterogeneity. Cancers. 2020;12. 10.3390/cancers12123742 PMC7764578 33322769

[9] Buss EC, Ho AD. Leukemia stem cells. Int J Cancer. 2011;129:2328–36. 21796620 10.1002/ijc.26318

[10] Hanekamp D, Cloos J, Schuurhuis GJ. Leukemic stem cells: identification and clinical application. Int J Hematol. 2017;105:549–57. 28357569 10.1007/s12185-017-2221-5

[11] Hope KJ, Jin L, Dick JE. Human acute myeloid leukemia stem cells. Arch Med Res. 2003;34:507–14. 14734090 10.1016/j.arcmed.2003.08.007

[12] Sarry JE, Murphy K, Perry R, Sanchez PV, Secreto A, Keefer C, et al. Human acute myelogenous leukemia stem cells are rare and heterogeneous when assayed in NOD/SCID/IL2Rgammac-deficient mice. J Clin Investig. 2011;121:384–95. 21157036 10.1172/JCI41495 PMC3007135

[13] Somervaille TC, Cleary ML. Identification and characterization of leukemia stem cells in murine MLL-AF9 acute myeloid leukemia. Cancer Cell. 2006;10:257–68. 17045204 10.1016/j.ccr.2006.08.020

[14] Wang X, Huang S, Chen JL. Understanding of leukemic stem cells and their clinical implications. Mol Cancer. 2017;16:2. 28137304 10.1186/s12943-016-0574-7 PMC5282926

[15] Bukowski K, Kciuk M, Kontek R. Mechanisms of multidrug resistance in cancer chemotherapy. Int J Mol Sci. 2020;21. 10.3390/ijms21093233 PMC7247559 32370233

[16] Zhang J, Gu Y, Chen B. Mechanisms of drug resistance in acute myeloid leukemia. Onco Targets Ther. 2019;12:1937–45. 30881045 10.2147/OTT.S191621 PMC6417008

[17] Neumann CA, Cao J, Manevich Y. Peroxiredoxin 1 and its role in cell signaling. Cell Cycle. 2009;8:4072–8. 19923889 10.4161/cc.8.24.10242 PMC7161701

[18] Egler RA, Fernandes E, Rothermund K, Sereika S, de Souza-Pinto N, Jaruga P, et al. Regulation of reactive oxygen species, DNA damage, and c-Myc function by peroxiredoxin 1. Oncogene. 2005;24:8038–50. 16170382 10.1038/sj.onc.1208821

[19] Nicolussi A, D’Inzeo S, Capalbo C, Giannini G, Coppa A. The role of peroxiredoxins in cancer. Mol Clin Oncol. 2017;6:139–53. 28357082 10.3892/mco.2017.1129 PMC5351761

[20] Ding C, Fan X, Wu G. Peroxiredoxin 1 - an antioxidant enzyme in cancer. J Cell Mol Med. 2017;21:193–202. 27653015 10.1111/jcmm.12955 PMC5192802

[21] Godfrey R, Arora D, Bauer R, Stopp S, Muller JP, Heinrich T, et al. Cell transformation by FLT3 ITD in acute myeloid leukemia involves oxidative inactivation of the tumor suppressor protein-tyrosine phosphatase DEP-1/ PTPRJ. Blood. 2012;119:4499–511. 22438257 10.1182/blood-2011-02-336446

[22] Wei W, et al. Identification of H7 as a novel peroxiredoxin I inhibitor to induce differentiation of leukemia cells. Oncotarget. 2016;7:3873–83. 26716647 10.18632/oncotarget.6763 PMC4826176

[23] Panieri E, Santoro MM. ROS homeostasis and metabolism: a dangerous liason in cancer cells. Cell Death Dis. 2016;7:e2253. 27277675 10.1038/cddis.2016.105 PMC5143371

[24] Hole PS, Zabkiewicz J, Munje C, Newton Z, Pearn L, White P, et al. Overproduction of NOX-derived ROS in AML promotes proliferation and is associated with defective oxidative stress signaling. Blood. 2013;122:3322–30. 24089327 10.1182/blood-2013-04-491944

[25] Prieto-Bermejo R, Romo-Gonzalez M, Perez-Fernandez A, Ijurko C, Hernandez-Hernandez A. Reactive oxygen species in haematopoiesis: leukaemic cells take a walk on the wild side. J Exp Clin Cancer Res. 2018;37:125. 29940987 10.1186/s13046-018-0797-0 PMC6019308

[26] Zhao RZ, Jiang S, Zhang L, Yu ZB. Mitochondrial electron transport chain, ROS generation and uncoupling (Review). Int J Mol Med. 2019;44:3–15. 31115493 10.3892/ijmm.2019.4188 PMC6559295

[27] Xian D, Lai R, Song J, Xiong X, Zhong J. Emerging perspective: role of increased ROS and redox imbalance in skin carcinogenesis. Oxid Med Cell Longev. 2019;2019:8127362. 31636809 10.1155/2019/8127362 PMC6766104

[28] Korswagen HC. Regulation of the Wnt/beta-catenin pathway by redox signaling. Dev Cell. 2006;10:687–8. 16740470 10.1016/j.devcel.2006.05.007

[29] Hayes JD, Dinkova-Kostova AT, Tew KD. Oxidative stress in cancer. Cancer Cell. 2020;38:167–97. 32649885 10.1016/j.ccell.2020.06.001 PMC7439808

[30] Zhou X, An B, Lin Y, Ni Y, Zhao X, Liang X. Molecular mechanisms of ROS-modulated cancer chemoresistance and therapeutic strategies. Biomed Pharmacother. 2023;165:115036. 37354814 10.1016/j.biopha.2023.115036

[31] Perillo B, Di Donato M, Pezone A, Di Zazzo E, Giovannelli P, Galasso G, et al. ROS in cancer therapy: the bright side of the moon. Exp Mol Med. 2020;52:192–203. 32060354 10.1038/s12276-020-0384-2 PMC7062874

[32] Wang J, Wong YK, Liao F. What has traditional Chinese medicine delivered for modern medicine?. Expert Rev Mol Med. 2018;20:e4. 29747718 10.1017/erm.2018.3

[33] Jafari R, Almqvist H, Axelsson H, Ignatushchenko M, Lundback T, Nordlund P, et al. The cellular thermal shift assay for evaluating drug target interactions in cells. Nat Protoc. 2014;9:2100–22. 25101824 10.1038/nprot.2014.138

[34] Chen B, Lee JB, Kang H, Minden MD, Zhang L. Targeting chemotherapy-resistant leukemia by combining DNT cellular therapy with conventional chemotherapy. J Exp Clin Cancer Res. 2018;37:88. 29690909 10.1186/s13046-018-0756-9 PMC5916833

[35] de Beauchamp L, Himonas E, Helgason GV. Mitochondrial metabolism as a potential therapeutic target in myeloid leukaemia. Leukemia. 2022;36:1–12. 34561557 10.1038/s41375-021-01416-w PMC8727299

[36] Suttana W, Mankhetkorn S, Poompimon W, Palagani A, Zhokhov S, Gerlo S, et al. Differential chemosensitization of P-glycoprotein overexpressing K562/Adr cells by withaferin A and Siamois polyphenols. Mol Cancer. 2010;9:99. 20438634 10.1186/1476-4598-9-99 PMC2873443

[37] Cui Q, Wang JQ, Assaraf YG, Ren L, Gupta P, Wei L, et al. Modulating ROS to overcome multidrug resistance in cancer. Drug Resist Updat. 2018;41:1–25. 30471641 10.1016/j.drup.2018.11.001

[38] Chou TC. Drug combination studies and their synergy quantification using the Chou-Talalay method. Cancer Res. 2010;70:440–6. 20068163 10.1158/0008-5472.CAN-09-1947

[39] Kulkarni U, Mathews V. Evolving chemotherapy free regimens for acute promyelocytic leukemia. Front Oncol. 2021;11:621566. 33718181 10.3389/fonc.2021.621566 PMC7947681

[40] Wang ZY, Chen Z. Acute promyelocytic leukemia: from highly fatal to highly curable. Blood. 2008;111:2505–15. 18299451 10.1182/blood-2007-07-102798

[41] Molica M, Mazzone C, Niscola P, de Fabritiis P. TP53 mutations in acute myeloid leukemia: still a daunting challenge?. Front Oncol. 2020;10:610820. 33628731 10.3389/fonc.2020.610820 PMC7897660

[42] Boyd AL, Lu J, Hollands CG, Alsostovar L, Murali S, Reid JC, et al. Leukemic progenitor compartment serves as a prognostic measure of cancer stemness in patients with acute myeloid leukemia. Cell Rep Med. 2023;4:101108. 37433297 10.1016/j.xcrm.2023.101108 PMC10394166

[43] Peng M, Huang Y, Zhang L, Zhao X, Hou Y. Targeting mitochondrial oxidative phosphorylation eradicates acute myeloid leukemic stem cells. Front Oncol. 2022;12:899502. 35574326 10.3389/fonc.2022.899502 PMC9100571

[44] Sallmyr A, Fan J, Datta K, Kim KT, Grosu D, Shapiro P, et al. Internal tandem duplication of FLT3 (FLT3/ITD) induces increased ROS production, DNA damage, and misrepair: implications for poor prognosis in AML. Blood. 2008;111:3173–82. 18192505 10.1182/blood-2007-05-092510

[45] Van Loenhout J, Peeters M, Bogaerts A, Smits E, Deben C. Oxidative stress-inducing anticancer therapies: taking a closer look at their immunomodulating effects. Antioxidants. 2020;9:1188. 33260826 10.3390/antiox9121188 PMC7759788

[46] Wang H, Gao Z, Liu X, Agarwal P, Zhao S, Conroy DW, et al. Targeted production of reactive oxygen species in mitochondria to overcome cancer drug resistance. Nat Commun. 2018;9:562. 29422620 10.1038/s41467-018-02915-8 PMC5805731

[47] Xu H, Zhao H, Ding C, Jiang D, Zhao Z, Li Y, et al. Celastrol suppresses colorectal cancer via covalent targeting peroxiredoxin 1. Signal Transduct Target Ther. 2023;8:51. 36732502 10.1038/s41392-022-01231-4 PMC9895061

[48] Bajor M, Zych AO, Graczyk-Jarzynka A, Muchowicz A, Firczuk M, Trzeciak L, et al. Targeting peroxiredoxin 1 impairs growth of breast cancer cells and potently sensitises these cells to prooxidant agents. Br J Cancer. 2018;119:873–84. 30287919 10.1038/s41416-018-0263-y PMC6189216

