# Supplementary material for: Targeting PRDX1 impairs acute myeloid leukemic blasts and stem cells by disrupting redox homeostasis
Source: Cell Death Dis. 2025 Aug 18;16(1):627. doi: 10.1038/s41419-025-07831-6 (PMC12361388; doi:10.1038/s41419-025-07831-6)
Supplement: Supplementary file 1 — Supplementary Data [file 41419_2025_7831_MOESM1_ESM.pdf]

Supplementary Data for

Targeting PRDX1 Impairs Acute Myeloid Leukemic Blasts and Stem Cells by Disrupting Redox Homeostasis

This PDF includes:

Figures S1 to S10

Tables S1, S2

Supplementary Figures

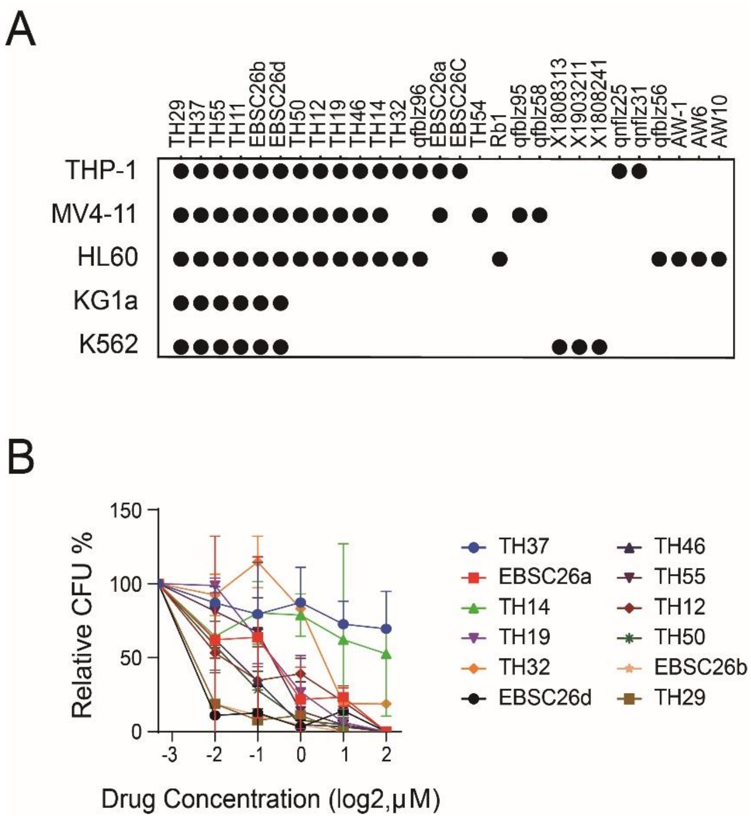

Figure S1. Drug Screening Results

(A) Inhibitory effects of TCM-derived compounds on colony formation in leukemia cells. Black dots indicate compounds with >50% efficacy against the specified leukemia cell types. (B) Counter-screening results showing the toxicity of selected compounds at the indicated concentrations on normal CD34+ cells (isolated from 2M-125C, Lonza, Basel, Switzerland) in colony formation assays. The relative average number of colonies ± SD from three independent cultures is shown. Distinct colors represent different compounds.

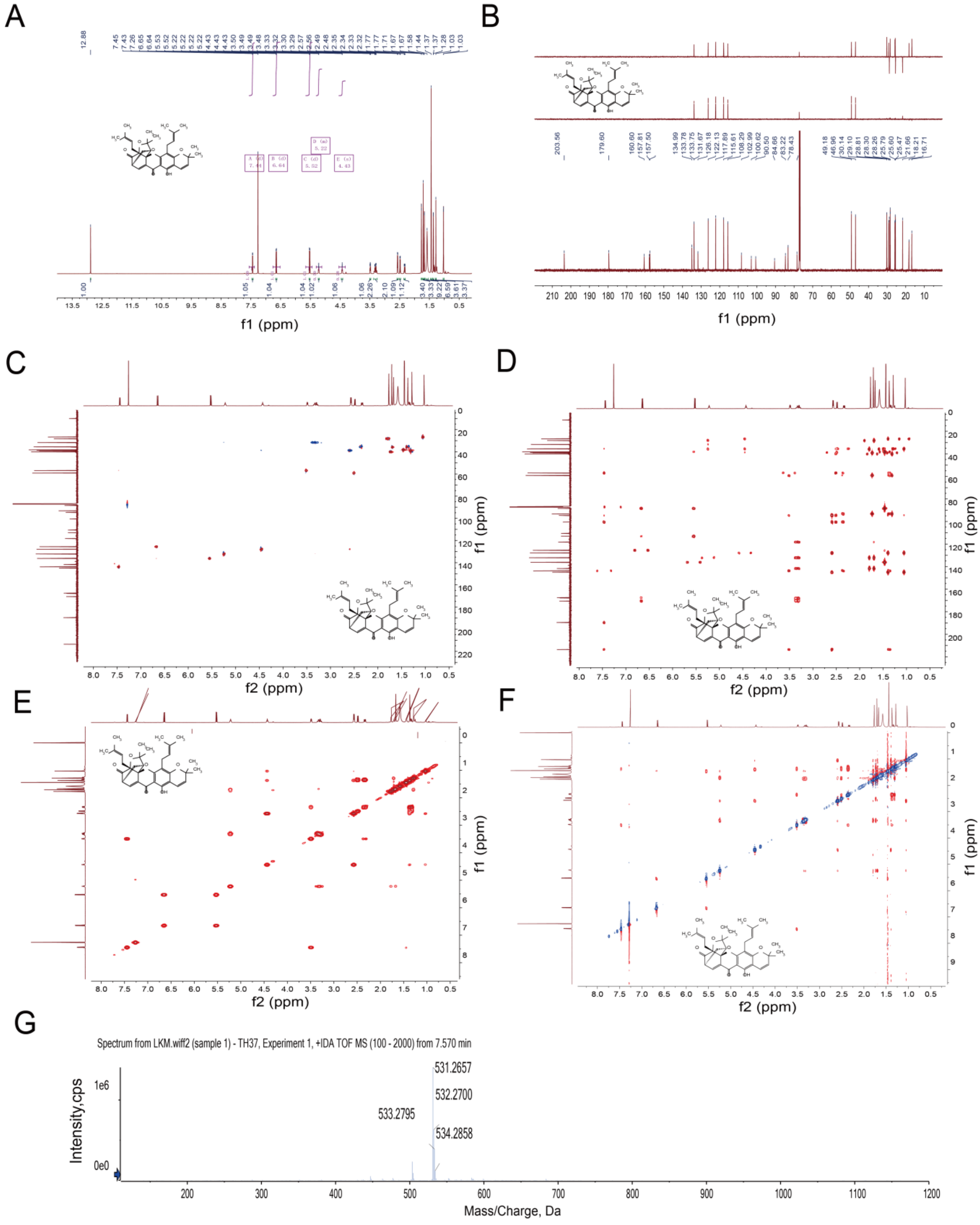

Hit	Formula	m/z	RDB	ppm	Found
1	$C_{33}H_{39}O_6$	531.2741	15	-15.8	NA/NA

Figure S2. Characterization of TH37

(A) ^{1}H NMR spectrum of TH37 in $CDCl_3$. (B) ^{13}C NMR and DEPT (Distortionless Enhancement by Polarization Transfer) spectra of TH37 in $CDCl_3$. (C) HSQC(Heteronuclear Single Quantum Coherence) spectrum of TH37 in $CDCl_3$. (D) HMBC spectrum of TH37 in $CDCl_3$. (E) ^{1}H–^{1}H COSY(Correlation Spectroscopy) spectrum of TH37 in $CDCl_3$. (F) ROESY(Rotating-Frame Overhauser Effect Spectroscopy) spectrum of TH37 in $CDCl_3$. (G) HRESIMS (High-Resolution Electrospray Ionization Mass Spectrometry) analysis confirmed the molecular formula as $C_{33}H_{39}O_6$, with the $[M+H]^+$ ion observed at m/z 531.2657 (calculated for $C_{33}H_{39}O_6$ $[M+H]^+$: 531.2741).

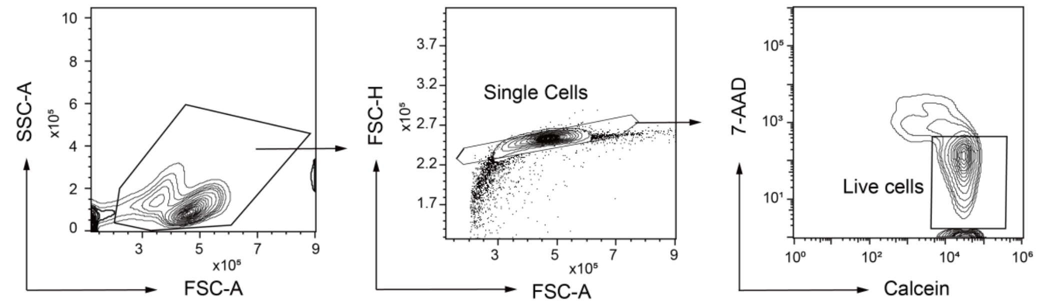

Figure S3. Gating strategy for live cells.

Live cells were identified using Calcein and 7-AAD staining in flow cytometry analysis.

A

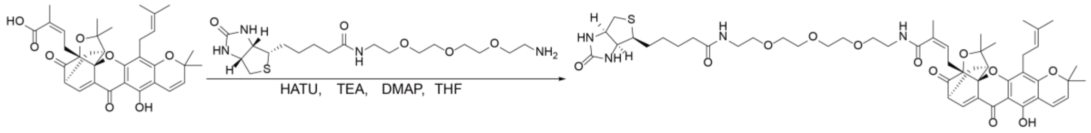

TH37 derivative

TH37-biotin

B

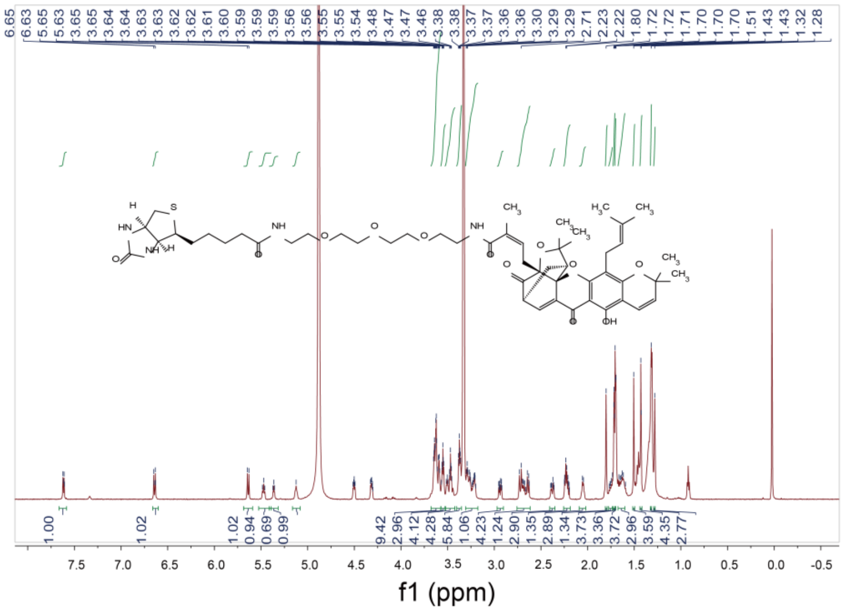

C

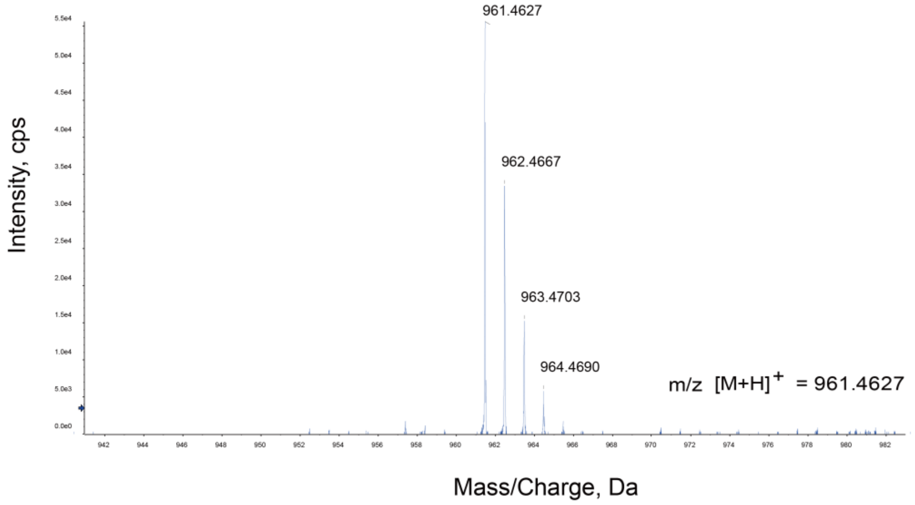

Hit	Formula	m/z	RDB	ppm	MS Rank	Found
1	$C_{51}H_{69}N_4O_{12}S$	961.46	20	0	1	NA/NA

Figure S4. Synthesis of TH37-biotin.

(A) Reaction scheme of synthesizing TH37-biotin. A solution of an oxidative derivative of TH37 (TH11, 5.6 mg) with equal biological potency, N-(2-(2-(2-(2-aminoethoxy)ethoxy)ethoxy)ethyl)-5-((3aS,4S,6aR)-2-oxohexahydro-1H-thieno[3,4-d]imidazol-4-yl)pentanamide (4.2 mg), triethylamine (TEA, 3 μL), 2-(7-azabenzotriazol-1-yl)-N,N,N',N'-tetramethyluronium hexafluorophosphate (HATU, 7.8 mg) and 4-dimethylaminopyridine (DMAP, 1.21 mg) in 5 mL tetrahydrofuran (THF) was stirred at room temperature. The reaction was monitored by thin layer charomatography (TLC) and when the reaction was completed, the reaction mixture was neutralized with saturated aq. NaCl. The resulting mixture was extracted with ethyl acetate and the organic layer was then washed with saturated aq. NaCl and dried over $MgSO_4$. After removal of the solvent under reduced pressure, the residue was purified by semi-preparative HPLC (YMC-Park-ODS, acetonitrile/H_2O = 80/20 containing0.05% TFA, flow rate: 3 mL/min) to yield compound TH37-biotin (3.1 mg, tR =8.3 min). (B) The ^1H NMR spectrum of TH37-biotin (600 MHz, DMSO-d6): δ 7.61 (dd, J = 6.9, 2.4 Hz, 1H), 6.64 (dd, J = 10.0, 2.5 Hz, 1H), 5.64 (d, J = 10.0 Hz, 1H), 5.47 (t, J = 7.6 Hz, 1H), 5.36 (t, J = 4.8 Hz, 1H), 5.12 (s, 1H), 4.55 – 4.45 (m, 1H), 4.32 (dd, J = 7.8, 4.5 Hz, 1H), 3.70 – 3.57 (m, 9H), 3.58 – 3.53 (m, 2H), 3.53 – 3.44 (m, 4H), 3.37 (td, J = 5.5, 2.3 Hz, 4H), 3.32 – 3.18 (m, 6H), 2.94 (ddd, J = 12.8, 5.0, 2.4 Hz, 1H), 2.76 – 2.62 (m, 4H), 2.42 – 2.34 (m, 2H), 2.22 (dt, J = 14.6, 7.1 Hz, 4H), 2.05 (d, J = 6.7 Hz, 1H), 1.80 (s, 3H), 1.78 – 1.72 (m, 1H), 1.72 (s, 3H), 1.70 (s, 3H), 1.68 – 1.58 (m, 1H), 1.51 (s, 3H), 1.43 (s, 3H), 1.32 (s, 3H), 1.28 (s, 3H). (C) The HRESIMS of TH37-biotin: m/z $[M+H]^+$ calculated for $C_{51}H_{69}N_4O_{12}S$ 961.4627, found: 961.4627.

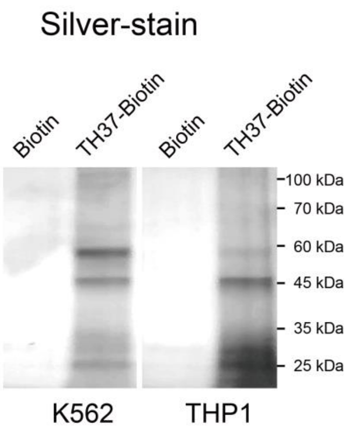

Figure S5. Separation of TH37-biotin captured proteins

Proteins captured by TH37-biotin or N3-biotin (control) were separated by SDS-PAGE and visualized using silver staining.

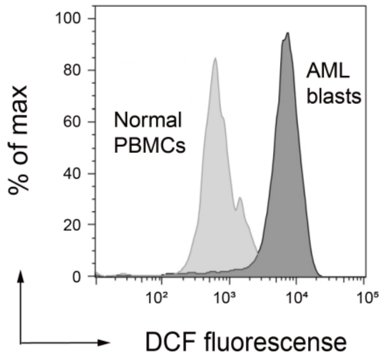

Figure S6. ROS levels in normal PBMCs (CD45+) and primary AML blasts.

ROS levels were compared between normal PBMCs (CD45+) and primary AML blasts (CD45 dim, SSC low) using flow cytometry analysis.

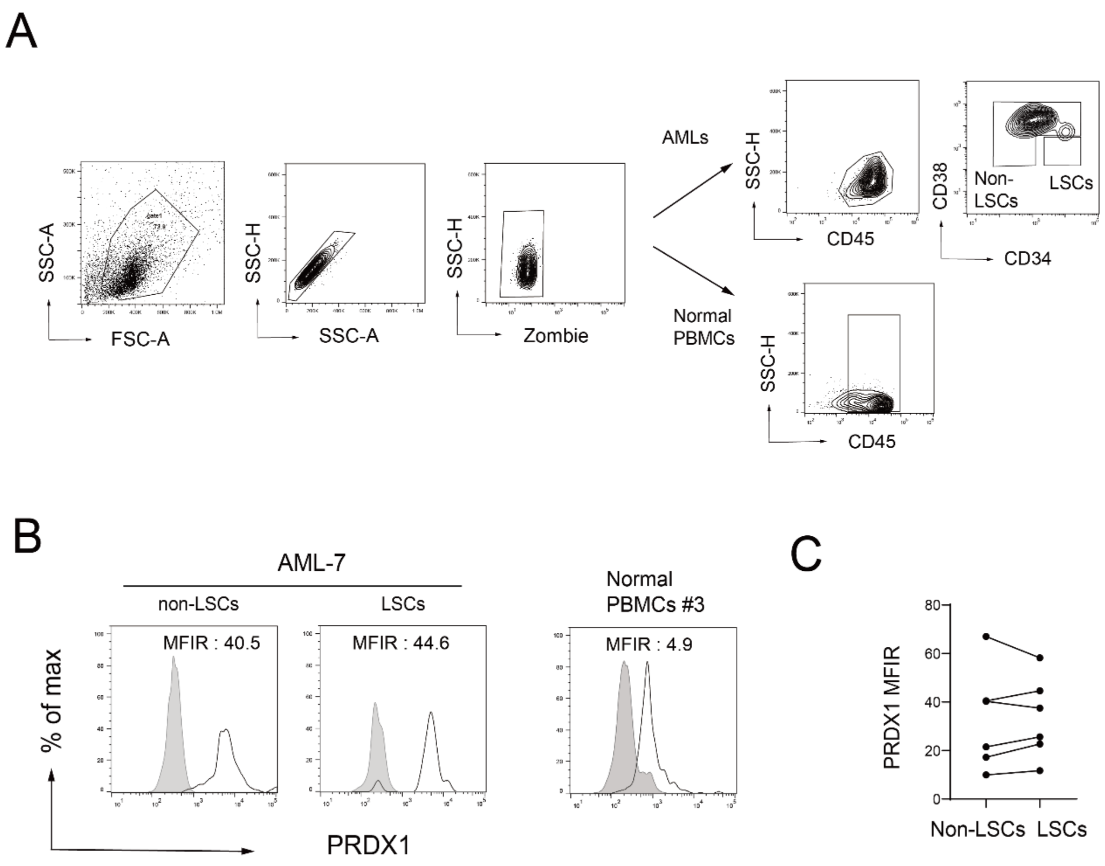

Figure S7. Intracellular staining of PRDX1 in LSCs

Intracellular PRDX1 staining in primary AML samples using Alexa Fluor 488-conjugated anti-human PRDX1 antibody. (A) Gating strategy (B) Representative histogram overlay demonstrating PRDX1 expression levels. (C) Comparative analysis of PRDX1 expression between LSCs (CD34+CD38-) (median=30.9) and non-LSC populations(median=31.5).

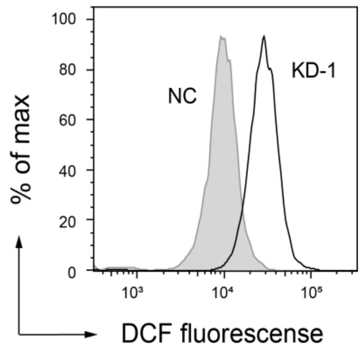

Figure S8. ROS level increased in PRDX1-KD AML cells

Stably PRDX1-KD(KD-1) or NC KG1a cells were stained with DCF for flow analysis.

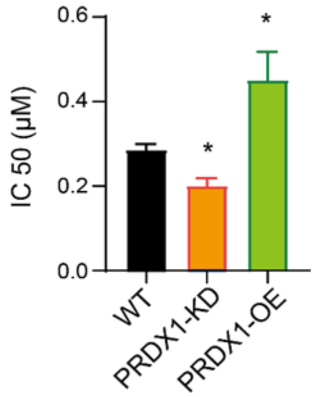

Figure S9. Overexpression of PRDX1 decreased sensitivity to TH37

IC50 values of TH37 in PRDX1-knockdown (PRDX1-KD, KD-1) or PRDX1-overexpressing (PRDX1-OE) KG1a cells were compared to those in wild-type (WT) control cells, as determined by the CCK-8 cell viability assay.

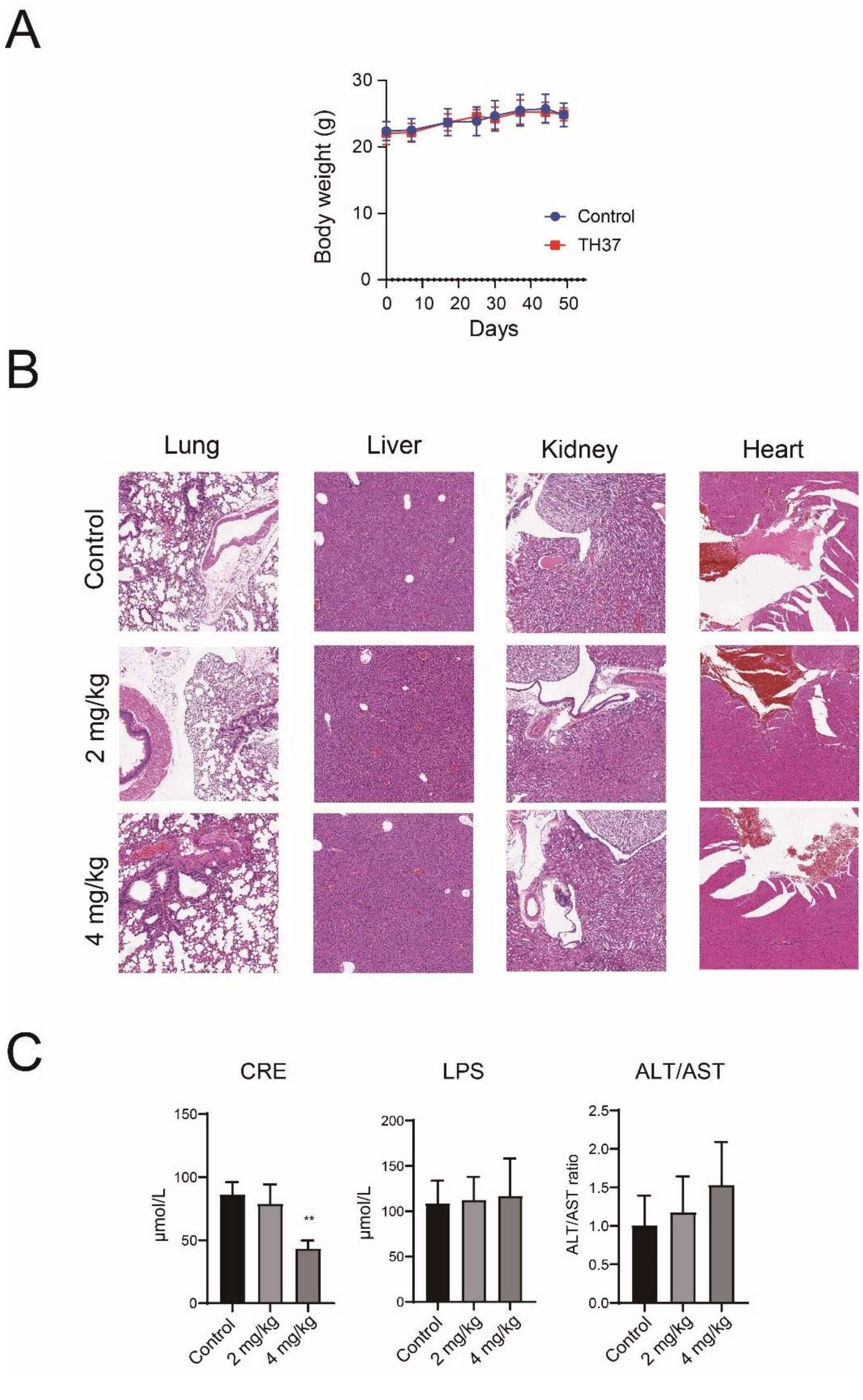

A
Body weight (g)
Days
Control
TH37
B
Lung
Liver
Kidney
Heart
Control
2 mg/kg
4 mg/kg
C
CRE
LPS
ALT/AST
μmol/L
ALT/AST ratio
**

Figure S10. Effects of TH37 on mice.

(A) Comparison of average body weight between TH37-treated and control groups at the indicated time points in the CDX experiment. (B-C) C57BL/6 wild-type mice were administered TH37 at specified doses (n = 3 per group) every two days and sacrificed after 28 days for tissue toxicity assessment. (B) Representative H&E staining results (C) Serum biochemical parameters measured at the end of the 28-day treatment period. Statistical significance was determined by Student's t test.

Supplementary Tables

Table S1. Cell line information

Cell line	Species	Origin	Cell bank / Supplier	Culture Medium
THP-1	Human	AML	NCACC	RPMI1640 + 10% FBS
K562	Human	CML	NCACC	RPMI1640 + 10% FBS
KG1a	Human	AML	ATCC	RPMI1640 + 10% FBS
MV4-11	Human	AML	NCACC	RPMI1640 + 10% FBS
HL60	Human	AML	NCACC	RPMI1640 + 10% FBS
K562-ADR	Human	CML	GeneChem inc. (Shanghai, China)	RPMI1640 + 10% FBS
Kasumi-1	Human	AML	ATCC	RPMI1640 + 10% FBS
293T	Human	Kidney	ATCC	DMEM + 10% FBS

NCACC, National Collection of Authenticated Cell Cultures (Shanghai, China)
ATCC, American Type Culture Collection

Table S2. Antibody information

Name	Supplier	Catalog No.	Application
Anti-Human CD45	BioLegend (San Diego, CA, USA)	982304	Flow
Anti-Human CD34	BioLegend (San Diego, CA, USA)	343616	Flow
Anti-Human CD38	BioLegend (San Diego, CA, USA)	303506	Flow
Anti-Mouse CD45	BD pharmingen (Milpitas, CA,USA)	561088	Flow
Anti-Human PRDX1	Thermo Fisher Scientific (Waltham, MA, USA)	PA5-27487	WB & Flow
Anti-Human GAPDH	Cell Signaling Technology (Boston, MA, USA)	2118	WB
