# Supplementary figures and images for: Targeting PRDX1 impairs acute myeloid leukemic blasts and stem cells by disrupting redox homeostasis
Source: Cell Death Dis. 2025 Aug 18;16(1):627. doi: 10.1038/s41419-025-07831-6 (PMC12361388; doi:10.1038/s41419-025-07831-6)

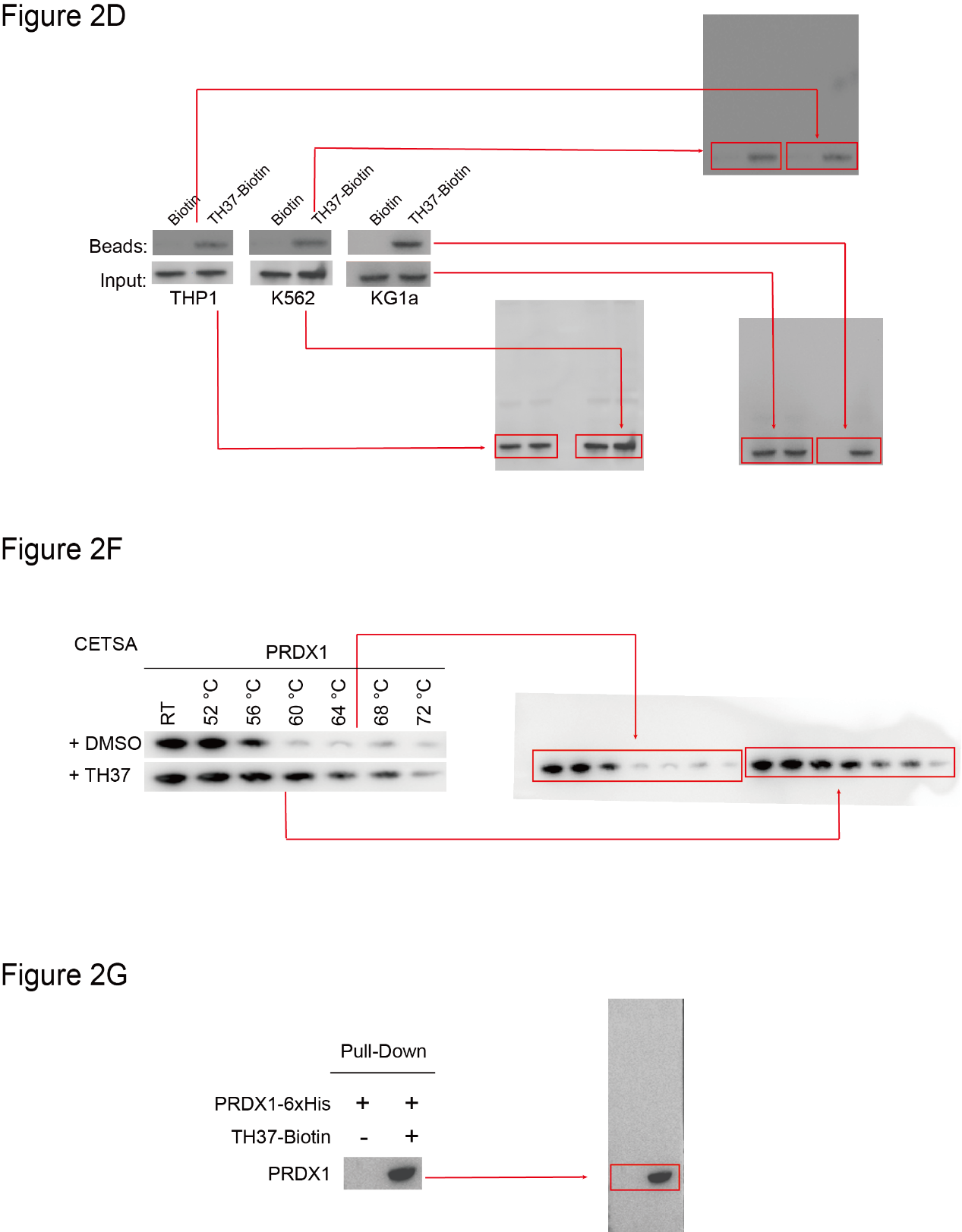


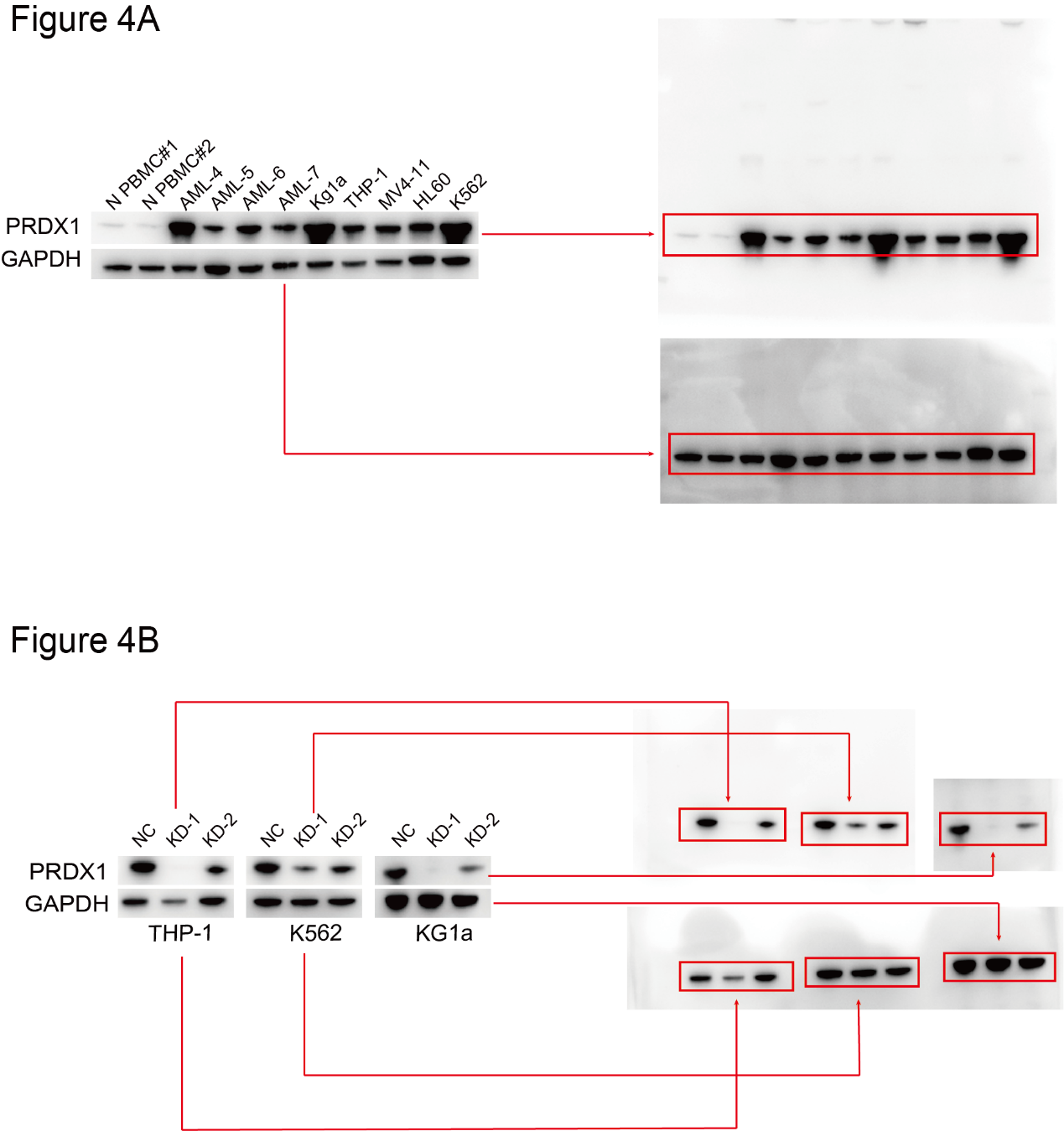

Supplement: Supplementary file 2 — Original WB data [file 41419_2025_7831_MOESM2_ESM.docx]
